# PDGF Receptors and Signaling Are Required for 3D-Structure Formation and Differentiation of Human iPSC-Derived Hepatic Spheroids

**DOI:** 10.3390/ijms24087075

**Published:** 2023-04-11

**Authors:** Syusaku Tsuzuki, Tomoyuki Yamaguchi, Takashi Okumura, Toshiharu Kasai, Yasuharu Ueno, Hideki Taniguchi

**Affiliations:** 1 Division of Regenerative Medicine, Center for Stem Cell Biology and Regenerative Medicine, The Institute of Medical Science, The University of Tokyo, Tokyo 108-0071, Japan; 2 Laboratory of Regenerative Medicine, Tokyo University of Pharmacy and Life Science, Tokyo 192-0392, Japan

**Keywords:** human iPSC, 3D culture, liver organoids, hepatic spheroids, platelet-derived growth factors (PDGFs), PDGF receptors

## Abstract

Human iPSC-derived liver organoids (LO) or hepatic spheroids (HS) have attracted widespread interest, and the numerous studies on them have recently provided various production protocols. However, the mechanism by which the 3D structures of LO and HS are formed from the 2D-cultured cells and the mechanism of the LO and HS maturation remain largely unknown. In this study, we demonstrate that *PDGFRA* is specifically induced in the cells that are suitable for HS formation and that PDGF receptors and signaling are required for HS formation and maturation. Additionally, in vivo, we show that the localization of PDGFRα is in complete agreement with mouse E9.5 hepatoblasts, which begin to form the 3D-structural liver bud from the single layer. Our results present that *PDGFRA* play important roles for 3D structure formation and maturation of hepatocytes in vitro and in vivo and provide a clue to elucidate the hepatocyte differentiation mechanism.

## 1. Introduction

In recent years, liver organoids (LO) or hepatic spheroids (HS) have attracted widespread interest due to their potential for use in basic research, pharmaceutical development, and regenerative medicine. In particular, human induced pluripotent stem cell-derived LO (hiPSC-LO) or HS (hiPSC-HS), which are produced using hepatocyte-like cells (HLCs) induced from hiPSCs, have advantages in ethics and availability. Therefore, numerous studies on hiPSC-LO or hiPSC-HS have been rapidly conducted and provided various production protocols for them [1,2,3,4,5,6,7]. For example, Wu et al. used hiPSC-HS generated from overgrown hepatic lineage cells in 2D-culture for their hiPSC-LO without Matrigel-embedding [3]. Ouchi et al. initially induced hiPSC-HS and then embedded them in Matrigel for 3D-culture of hiPSC-LO differentiation [4]. Although these protocols are quite different, it is common that HLC on the 2D dish initially goes through HS, which are simple 3D structures without organ-like structures, then eventually forms LO. Thus, HS formation is likely necessary for LO production. However, the mechanism by which the 3D structures of HS are spontaneously formed from the 2D-cultured HLC and the mechanism of maturation of the HLCs composing the HS remain largely unknown. Similarly, the details of the molecular mechanisms by which liver buds are formed during in vivo liver development are largely unexplained.

It has been reported that hiPSC-derived hepatic endoderm (HE) defined by Kulkeaw et al. was able to form LO within Matrigel; however, further-differentiated AFP-positive hepatoblasts and ALB-positive hepatocytes were not able to form LOs [6]. Likewise, Takebe et al. have shown that hiPSC-derived liver buds (hiPSC-LB) were appropriately produced from HE in their protocol, whereas cells shifted from the HE for only ±2 culture days were not able to appropriately assemble into hiPSC-LB [2]. As in these reports, it seems that only specific cells differentiated between hiPSCs and mature HLCs are capable of formation and maturation of LO or HS; however, it remains unclear what factors determine the characteristics of these cells.

Platelet-derived growth factors (PDGFs) and receptors (PDGFRs) were originally identified as growth factors that act on mesenchymal cells (MC) and their counterparts. However, it is known that PDGFs and PDGFRs have many roles in various biological processes such as embryology, physiology, and pathology [8,9,10,11]. PDGFRs have in common with the hepatocyte growth factor (HGF) receptor (MET) that they belong to the receptor tyrosine kinase family and promote AKT signaling. Although HGF is well known to act on hepatocyte differentiation and proliferation, the action of PDGF on hepatocytes is largely unknown. Additionally, in liver development, it has been shown that the protein levels of PDGFRα and PDGFRβ were transiently increased in mouse fetal livers of E10 to 11 [12,13]. However, little is known about how PDGFRs function in the immature hepatic progenitor around E10 to 11.

This study aimed to identify the factors that determine the success of HS formation and maturation. This study demonstrates that PDGF receptors and signaling are required for HS formation and maturation. Furthermore, we investigate the relationship between PDGF and in vivo liver development by expression and localization analysis.

## 2. Results

**Figure 1:**
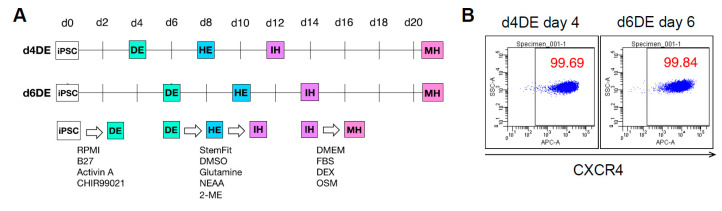

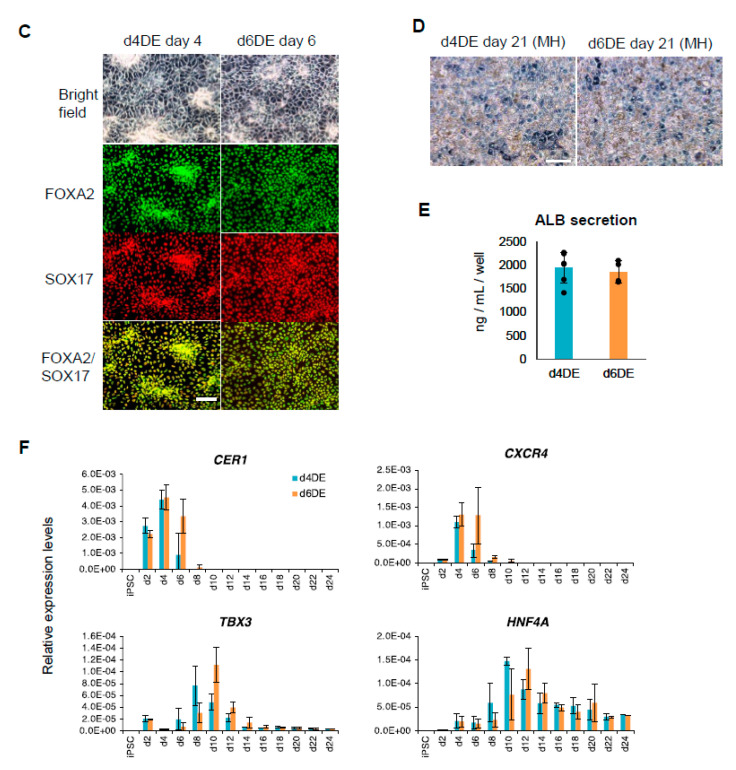
Details of d4DE and d6DE protocols. (**A**) Schematic overview of d4DE and d6DE protocol. d: day, DE: definitive endoderm, HE: hepatic endoderm, IH: immature hepatocyte, MH: mature hepatocyte. See also Materials and Methods. (**B**) Flow cytometric analysis of CXCR4. The numbers represent the positive rate (%). (**C**) Immunostaining of the DE markers, FOXA2 and SOX17. Scale bar, 100 μm. (**D**) Morphological representation of MH. Scale bar, 200 μm. (**E**) Albumin secretion of each MH on day 21. The bars represent mean ± SD. (*n* = 4 to 6). (**F**) Time course analysis of gene expression by qRT-PCR. Normalization is performed using the expression level of 18S rRNA. Data are represented as mean ± SD. (*n* = 3 to 6). See also Figure S1.

At first, to investigate which cell types are suitable for HS formation, two different differentiation protocols for generating HLC were used to examine HS-forming ability over time. In these HLC induction protocols, hiPSCs differentiate through definitive endoderm (DE), HE, and immature hepatocyte, and eventually mature hepatocyte (MH) [14]; however, the time to DE induction differs between 4 days and 6 days in the d4DE and d6DE protocols, respectively (Figure 1A). Comparing these two protocols, on both day 4 and day 6, the positivity rate of CXCR4, which is frequently used as a DE marker, exceeded 99.5% (Figure 1B). Furthermore, almost all of the cells were positive for SOX17 and FOXA2 by immunostaining (Figure 1C). Additionally, the morphologies of MH in the d4DE and d6DE protocol were almost identical (Figure 1D); enzyme-linked immunosorbent assay (ELISA) of albumin and quantitative reverse transcription polymerase chain reaction (qRT-PCR) analyses of hepatocyte markers showed that d4DE-MH and d6DE-MH have similar properties (Figure 1E and Figure S1). In contrast, time course expression analyses of DE markers (*CER1, CXCR4*) and HE markers (*TBX3, HNF4A*) showed that there was a two-day gap in the progression of differentiation between d4DE and d6DE protocols around day 6 to 14 (Figure 1F).

**Figure 2:**
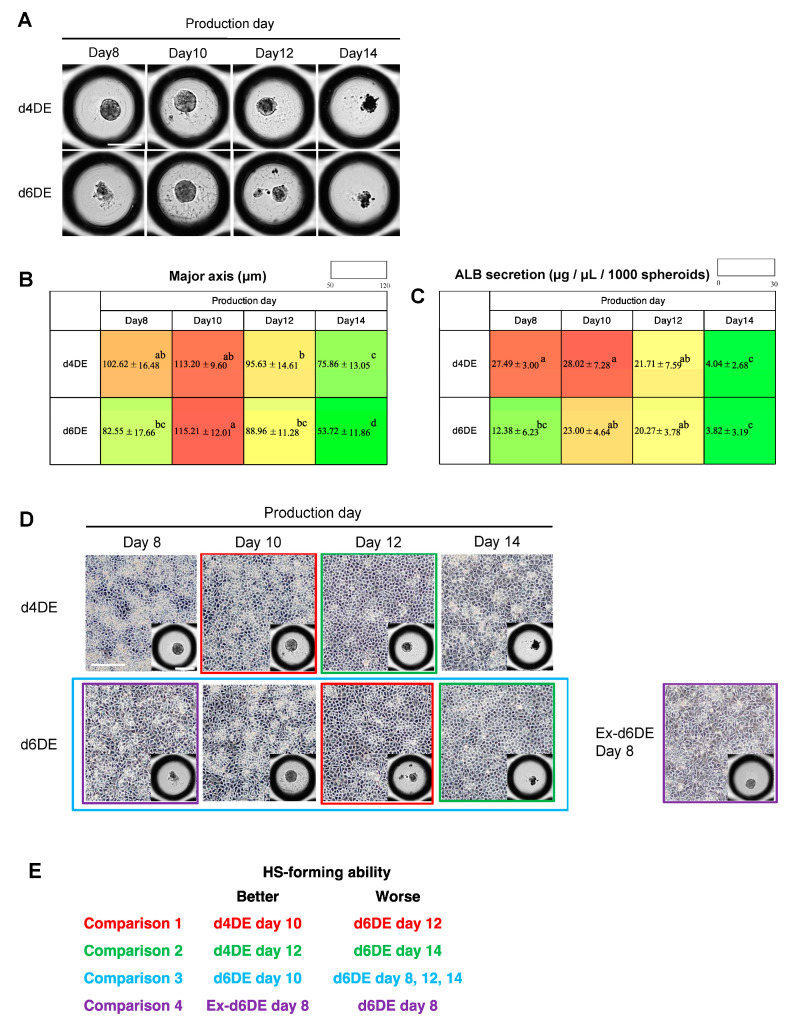
Comparison of HS generated at multiple timepoints in d4DE and d6DE protocols. (**A**) HS generated on day 8, 10, 12, and 14 in d4DE and d6DE protocols. Scale bar, 200 μm. (**B**,**C**) Major axis (**B**) and ALB secretion (**C**) of each HS at endpoint (total 21 days). Data are represented as mean ± SD. Numerical data with the different letters are significantly different according to one-way ANOVA with post-hoc Tukey HSD test (*p* < 0.05). (*n* = 10 to 11 in (**B**), *n* = 3 in (**C**)). (**D**) Morphological representation of cells on the 2D dish used for HS production. Colored outlines correspond to Comparison 1 to 4 in (**E**). Ex-d6DE: exceptional d6DE day 8, see also Figure S2. Scale bar, 200 μm. (**E**) Cells with better or worse HS-forming ability in Comparison 1 to 4. The colors of letters correspond to colored outline in (**D**). See also Figures S2 and S3.

We next produced HS on days 8, 10, 12, and 14 using both the d4DE and d6DE protocols (Figure 2A). These HS were cultured to the equivalent MH (for a total of 21 days), and their size and ALB secretion were measured. In the d6DE protocol, HS produced on day 10 had a morphologically well-assembled appearance, were large, and secreted the most amount of ALB than other HS timepoints (Figure 2A,C). Therefore, day 10 was the best timepoint for HS formation in the d6DE protocol. In contrast, under the d4DE protocol, HS produced on days 8, 10, and 12 exhibited sufficient morphology (Figure 2A). Furthermore, the size and ALB secretion of HS produced on days 8 and 12 were comparable to those of HS produced on day 10 in d4DE protocol (Figure 2B,C). These results suggest that the range of HS formation timing in the d4DE protocol was wider compared to the d6DE protocol.

Based on these observations, we attempted to explore specific factors that are required for HS formation and maturation by comparing the gene expression profiles of cells with the same differentiation stage but different spheroid-forming abilities. The gene expression of each cell used to produce HS (Figure 2D) was comprehensively analyzed by microarray. Using the datasets, we compared d4DE day 10 with d6DE day 12 (Comparison 1) and d4DE day 12 with d6DE day 14 (Comparison 2) (Figure 2D,E), which are combinations of cells with the same differentiation stage but different spheroid-forming ability. In addition, to explore genes involved in HS formation from multiple viewpoints, the gene expression profile of d6DE day 10 cells was compared with the group of those of d6DE day 8, 12, and 14 cells (Comparison 3) (Figure 2D,E). Moreover, when HS production on days 8, 10, 12, and 14 were repeated, exceptional d6DE day 8 (Ex-d6DE day 8) cells that formed well-assembled HS appeared only once in nine attempts (Figure S2). The Ex-d6DE day 8 was also compared with the normal d6DE day 8 (Comparison 4) (Figure 2D,E).

In each comparison, we screened genes which had expression levels that were more than two times higher (fold change > 1) in the cells that formed better HS than the cells forming worse HS (Table S1). Then, genes that encode proteins localized to the cell membrane were selected from the screened genes because it is assumed that cell–cell interaction is important for the 3D-structure formation of HS. Among the six genes found (Table S1), two transporter genes, *SLC39A4* and *SLCA11*, were eliminated because it appeared that they were not directly related to cell–cell interaction. Therefore, we focused on *PDGFRA*, *SEMA7A*, *EDARADD*, and *TMEM37* and verified the accuracy of the microarray analysis by qRT-PCR. Although only the *EDARADD* qRT-PCR result in Comparison 1 was inconsistent with the microarray results, agreements between qRT-PCR and microarray analysis were obtained in all other conditions (Figure S3).

**Figure 3:**
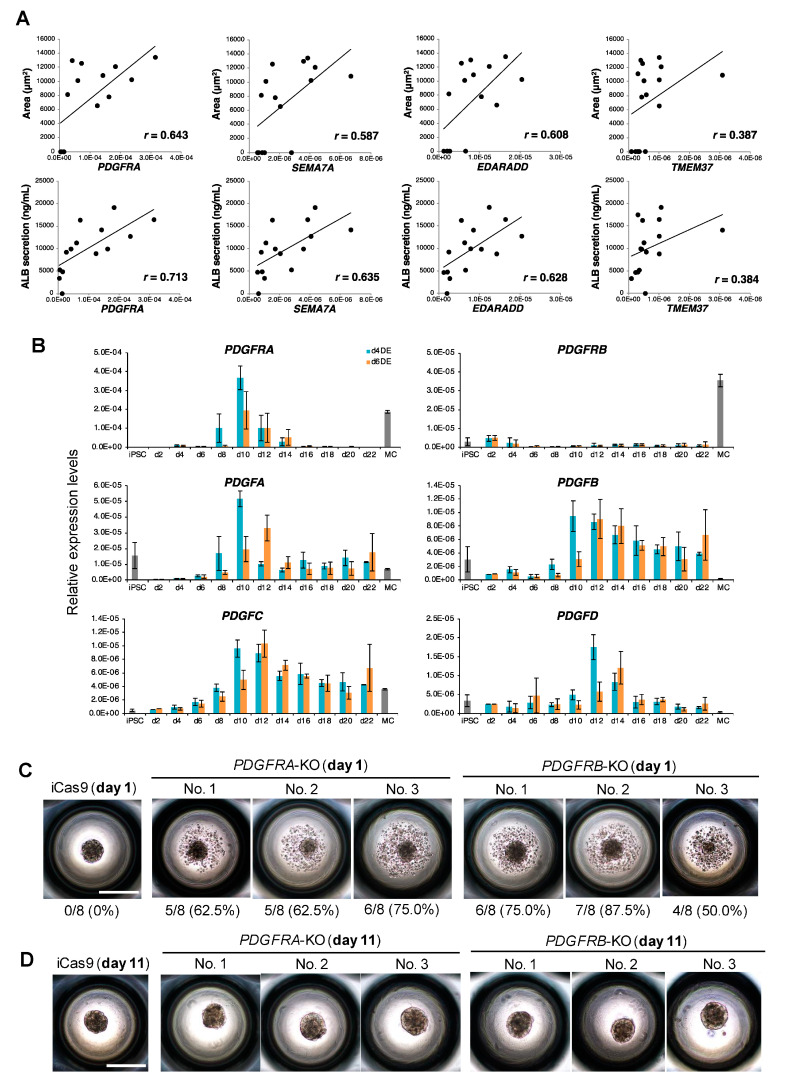
Details of d4DE and d6DE protocols. (**A**) Plots of the four genes’ expression levels against the size or ALB secretion amounts of each HS. Normalization of gene expression levels is performed using the expression level of 18S rRNA. Each area of HS was calculated by automated microscopy system. Each ALB secretion indicates the value per 600 HS. The *r* represents the correlation coefficient. (**B**) Time course analyses of mRNA levels of PDGFRs and PDGFs during differentiation. Normalization of gene expression levels is performed using the expression level of 18S rRNA. Data are represented as mean ± SD. (*n* = 3). (**C**,**D**) Morphologies of HS in iCas9, *PDGFRA*-KO, and *PDGFRB*-KO cell lines on day 1 (**C**) and day 11 (**D**) after production. Each percentage represents HS formation failure rate. Scale bar, 200 μm. See also Figures S4 and S5.

To investigate relationships among the four genes, HS formation, and maturation, expression levels of the four genes in d4DE and d6DE day 8, 10, 12, and 14 cells were plotted against the size or ALB secretion amounts of each HS (Figure 3A). Expression levels of *PDGFRA* displayed the strongest correlation with both HS size and ALB secretion in these plots (Figure 3A). Accordingly, we considered *PDGFRA* a candidate for the HS-forming factor and analyzed its expression pattern in more detail. The time course expression analysis showed that the peak of *PDGFRA* is day 10 in both the d4DE and d6DE protocols, which was consistent with the best timepoint of HS formation (Figure 3B). *PDGFRA*, which is well known as a marker of MC, was also highly expressed in iPSC-derived MC (Figure 3B). The expression levels of *PDGFRA* in d4DE and d6DE day 10 were comparable with or more than those in MCs (Figure 3B). These results suggest that *PDGFRA* might transiently play a role in some biological mechanisms in the process of HLC differentiation. In contrast, *PDGFRB* expression was very low during HLC differentiation compared to iPSC-derived MCs, suggesting that *PDGFRB* does not affect HLC differentiation (Figure 3B). Flow cytometry analysis also presented the highest positive rates of PDGFRα on day 10 in both the d4DE and d6DE protocols, as well as the qRT-PCR analysis (Figure S4). The d4DE protocol had a longer period of high PDGFRα-positive rates than the d6DE protocol, which was similar to the allowable range of HS formation timing in the d4DE protocol being longer than the d6DE protocol (Figure 2B–D). To investigate whether PDGF signaling can be activated when *PDGFRA* displays the expression peak, expression patterns of all types of PDGF were also analyzed. The analyses showed that expression peaks of *PDGFA*, *PDGFB*, and *PDGFC* were detected on day 10 in the d4DE protocol and on day 12 in the d6DE protocol and that only the expression peak of *PDGFD* was delayed for two days in both d4DE and d6DE protocol (Figure 3B). It has been known that PDGF dimers PDGFAA, PDGFAB, PDGFBB, and PDGFCC ligands interact with PDGFRαα homodimer. However, only PDGFDD does not interact with it [9]. These results suggested that PDGF signaling could be stimulated on days 10 to 12 with autocrine via at least PDGFRαα.

To examine whether *PDGFRA* is required for HS formation and maturation, we prepared *PDGFRA*-knockout (KO) hiPSC lines. We also prepared *PDGFRB*-KO hiPSC lines because PDGFRα interacts with PDGFRβ to form PDGFRαβ hetero-dimer. *PDGFRA*- or *PDGFRB*-KO hiPSC lines were generated by employing the CRISPR/Cas9 system as previously described [15,16] and were validated by sequence of the genome and flow cytometry analysis (Figure S5). In PDGFRB-KO cell lines, all picked up colonies had the same deletion pattern because all bases between two gRNA target sites were deleted (Figure S5). It was assumed that the frameshift did not occur because the deletion length was 183 bases (Figure S5). However, *PDGFRB*-KO was confirmed at the protein level by flow cytometry analysis, probably due to the substantially long base deletion (Figure S5).

*PDGFRA*-KO hiPSC lines (No. 1–3), *PDGFRB*-KO hiPSC lines (No. 1–3), and Cas9-introduced hiPSC line (iCas9) as control were differentiated in the d6DE protocol and were used to produce HS on day 10. Each HS of *PDGFRA*-KO lines and *PDGFRB*-KO lines displayed numerous non-aggregated cells on day 1 after HS production (Figure 3C). The failure of 3D-structure formation on day 1 was observed four to seven times out of eight trials (50%–87.5%) (Figure 3C). It was unexpected that failure of HS formation was induced in not only *PDGFRA*-KO but also *PDGFRB*-KO cell lines because the expression pattern of *PDGFRB* did not show a peak at day 10, unlike *PDGFRA* in the differentiation process of HLC (Figure 3B). These results imply that both *PDGFRA* and *PDGFRB* contribute to HS formation. However, by culturing the HS of *PDGFRA*-KO and *PDGFRB*-KO lines for 11 days (until the equivalent of MH, total of 21 days), their morphologies were improved and exhibited as well as iCas9 control HS (Figure 3D). This result suggests a reaggregation mechanism independent of first cell aggregation. Indeed, HS viabilities on day 11 of *PDGFRA*-KO and *PDGFRB*-KO lines were comparable to that of the iCas9 line (Figure S4). There was no significant difference in ALB secretion levels and expression levels of hepatocyte marker genes (*ALB, OTC, ARG1, CPS1*, and *SCD*) among *PDGFRA*-KO, *PDGFRB*-KO, and iCas9 HS (Figure S4).

**Figure 4:**
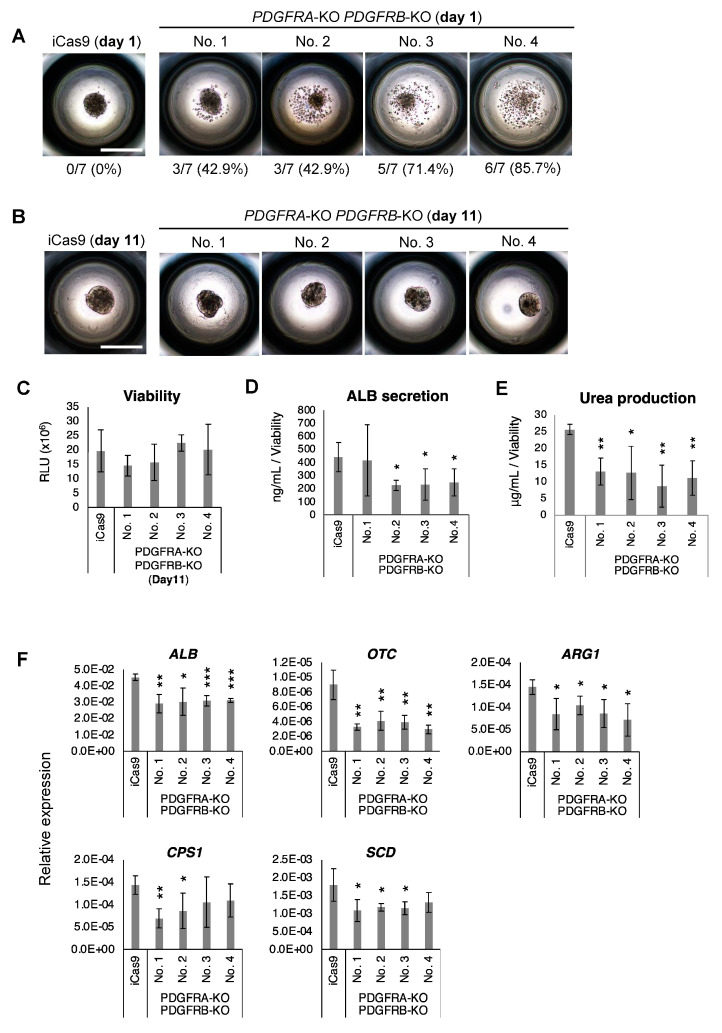
The phenotype analyses of *PDGFRA/B* double KO cell lines in HS. (**A**,**B**) Morphologies of HS in iCas9 and *PDGFRA/B* double KO cell lines on day 1 (**A**) and day 11 (**B**) after production. Each percentage represents HS formation failure rate. Scale bar, 200 μm. (**C**) Cell viability within HS by measuring intracellular ATP levels. The bars represent mean ± SD. (*n* = 5). RLU: relative luminescent unit. (**D**) Albumin secretion per cell viability of each HS on day 11 after production. The bars represent mean ± SD. (*n* = 5; * *p* < 0.05). (**E**) Urea production per cell viability of each HS on day 11 after production. The bars represent mean ± SD. (*n* = 5; * *p* < 0.05; ** *p* < 0.01). (**F**) Expression levels of hepatocyte marker genes in each HS on day 11 after production. Normalization is performed using the expression level of 18S rRNA. The bars represent mean ± SD. (*n* = 5; * *p* < 0.05; ** *p* < 0.01; *** *p* < 0.001). See also Figure S7.

To verify whether *PDGFRA* and *PDGFRB* (*PDGFRA/B*) double KO cell lines display severe additive phenotype compared with the single *PDGFRA*-KO or *PDGFRB*-KO cell lines, *PDGFRA/B* double KO cell lines (No. 1–4) were generated (Figure S6) and used for HS formation in the same way. Unexpectedly, the rates of HS formation failure on day 1 in the *PDGFRA/B* double KO cell lines were 42.9%–85.7%; there was little difference compared with the single *PDGFRA*-KO or *PDGFRB*-KO cell lines (Figure 4A). These results imply that there may be other factors supporting cell aggregation in HS formation. As well as single *PDGFRA*-KO or *PDGFRB*-KO cell lines, *PDGFRA/B* double KO HS were cultured for 11 days, and their HS were visibly almost identical to iCas9 control HS (Figure 4B). There was no significant difference in the viability among *PDGFRA/B* double KO HS and iCas9 HS (Figure 4C). Nevertheless, ALB secretion levels and the amount of urea resulting from NH_4_Cl metabolism per cell viability were significantly reduced in most *PDGFRA/B* double KO HS (Figure 4D,E). Moreover, expression levels of hepatocyte marker genes in the almost HS of *PDGFRA/B* double KO cell lines were significantly downregulated compared with those of iCas9 control (Figure 4F). These phenotypes were not observed in single *PDGFRA*-KO and *PDGFRB*-KO lines, but only emerged in *PDGFRA/B* double KO lines. In contrast, there was no significant difference among 2D-cultured MH derived from *PDGFRA/B* double KO lines and iCas9 lines in the hepatocyte marker genes’ expression, ALB secretion, and urea production levels (Figure S7). These results indicated that *PDGFRA* and *PDGFRB* were involved in the establishment of HS 3D-structure and the maturation of HLC only in the case of 3D-cultured HS.

**Figure 5:**
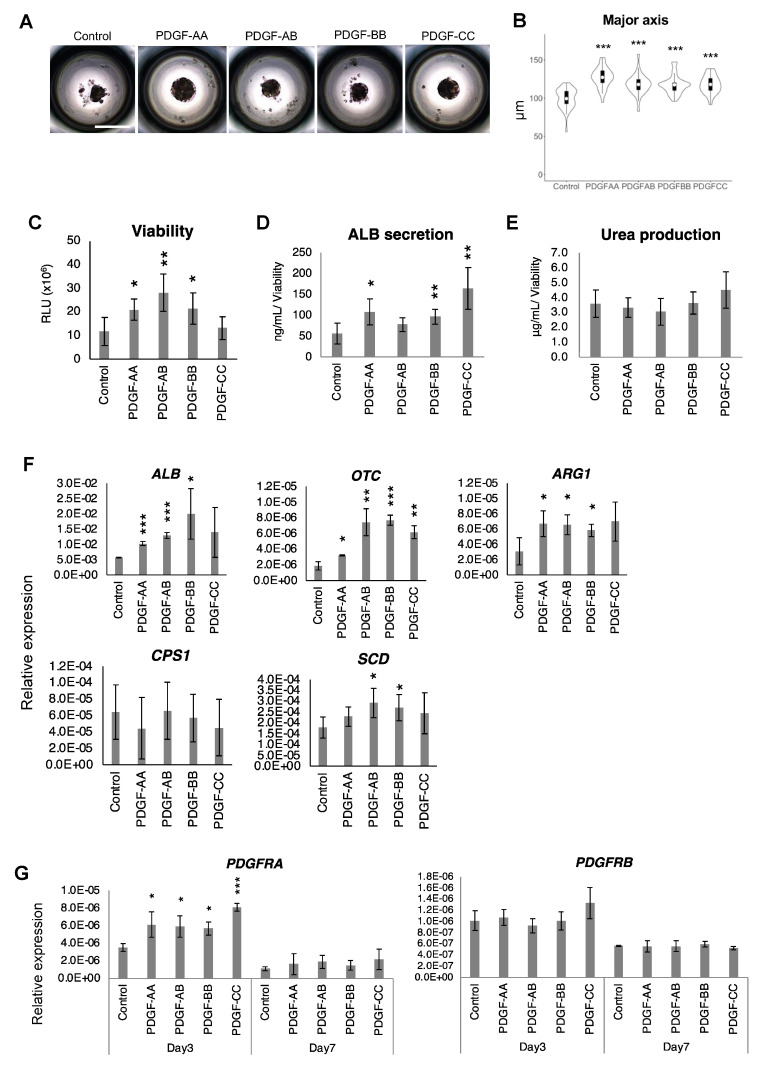
Evaluation of PDGFs’ effect on HS produced from d4DE day 14 cells. (**A**) Morphologies of mock control and each PDGF-treated HS on day 7 after production. Scale bar, 200 μm. (**B**) Violin plot of major axis length of mock control and each PDGF-treated HS on day 7. (*n* = 29 to 33; *** *p* < 0.001) (**C**) Cell viability within HS by measuring intracellular ATP levels in each HS. The bars represent mean ± SD. (*n* = 5; * *p* < 0.05; ** *p* < 0.01). RLU: relative luminescent unit. (**D**) Albumin secretion per cell viability in each HS. The bars represent mean ± SD. (*n* = 3; * *p* < 0.05; ** *p* < 0.01). (**E**) Urea production per cell viability in each HS. The bars represent mean ± SD. (*n* = 3). (**F**) Expression levels of hepatocyte marker genes in each HS. Normalization is performed using the expression level of 18S rRNA. The bars represent mean ± SD. (*n* = 3 to 5; * *p* < 0.05; ** *p* < 0.01; *** *p* < 0.001). (**G**) Expression levels of PDGFR genes of mock control and each PDGF-treated HS on day 3 and 7 after production. Normalization is performed using the expression level of 18S rRNA. The bars represent mean ± SD. (*n* = 3). See also Figure S8.

Because it was shown that PDGF receptors are required for the differentiation and maturation of HLC in 3D-cultured HS, it can be assumed that PDGF signaling via PDGF receptors plays a role in them. Thus, we examined whether PDGF signals improve differentiation, maturation, and 3D-structure formation of HS by treatment of PDGF-AA, -AB, -BB, and -CC when producing HS. HS were prepared using d4DE day 14 cells, which do not adequately assemble into HS by themselves and were treated with the PDGFs from the day of production to the endpoint equivalent to MH (for 7 days). In all PDGF-AA, -AB, -BB, and -CC treatment groups, HS morphology was considerably improved (Figure 5A), the size was significantly increased (Figure 5B), and the viability was also increased compared with mock control (Figure 5C). The levels of ALB secretion per cell viability were also significantly increased in the PDGF-AA, -BB, and -CC treatment groups (Figure 5D), although the urea production per cell viability of PDGFs treatment groups and mock control groups were almost the same level (Figure 5E). In addition, expression levels of hepatocyte marker genes *ALB, OTC, ARG1*, and *SCD* were also significantly increased in many cases of PDGF-AA, -AB, -BB, and -CC treatment groups (Figure 5F). These results indicated that PDGF signaling could promote both 3D-structure formation and maturation of HLC in the HS. Next, we evaluated that expression of *PDGFRA* and *PDGFRB* are responsive to PDGFs in the HS derived from d4DE day 14 cells. Although expression levels of *PDGFRA* declined at 7 days after HS production like the off-peak period under the 2D-culture condition, on day 3, *PDGFRA* was significantly induced by PDGF-AA, -AB, -BB, and -CC (Figure 5G). This result implies that PDGFs affect not only the enhancement of PDGF signaling but also the positive feedback loop by induction of *PDGFRA*. This might be a factor in dramatically promoting size and HLC maturation. In contrast, the effects of PDGFs on *PDGFRB* expression levels were not detected at all (Figure 5G). This result suggested that the pathway via PDGFRα is the mainstream in PDGF signaling of the HS. Furthermore, in the MH under 2D-culture conditions, treatments of PDGF-AA, -BB, and -CC significantly increased viability but had no effect on expression levels of hepatocyte marker and PDGF receptor genes (Figure S8). Therefore, it was confirmed that the promotion of hepatocyte maturation by PDGFs is specific to HLC in 3D-cultured HS.

To investigate whether PDGFs also improve the size and maturation level of appropriately produced HS, we similarly administered PDGFs to HS built from d6DE day 10 cells. Each HS size was significantly increased in all PDGF-AA, -AB, -BB, and -CC treatment groups, and the viabilities were also significantly increased in the PDGF-AB, -BB treatment group (Figure S9). However, there was little difference between PDGFs treatment groups and mock control in the amount of ALB secretion and urea production per cell viability (Figure S9). Expression analysis of hepatocyte marker genes showed that PDGF-AB and -BB treatment significantly increased *OTC* levels. Only PDGF-BB treatment significantly increased *CPS1* levels (Figure S9). The effect of PDGFs on HS of d6DE day 10 cells seemed mild compared to the effect on those of d4DE day 14 cells, which might be due to high expression levels of *PDGFRA* and *PDGFs* originally contained in d6DE day 10 cells.

**Figure 6:**
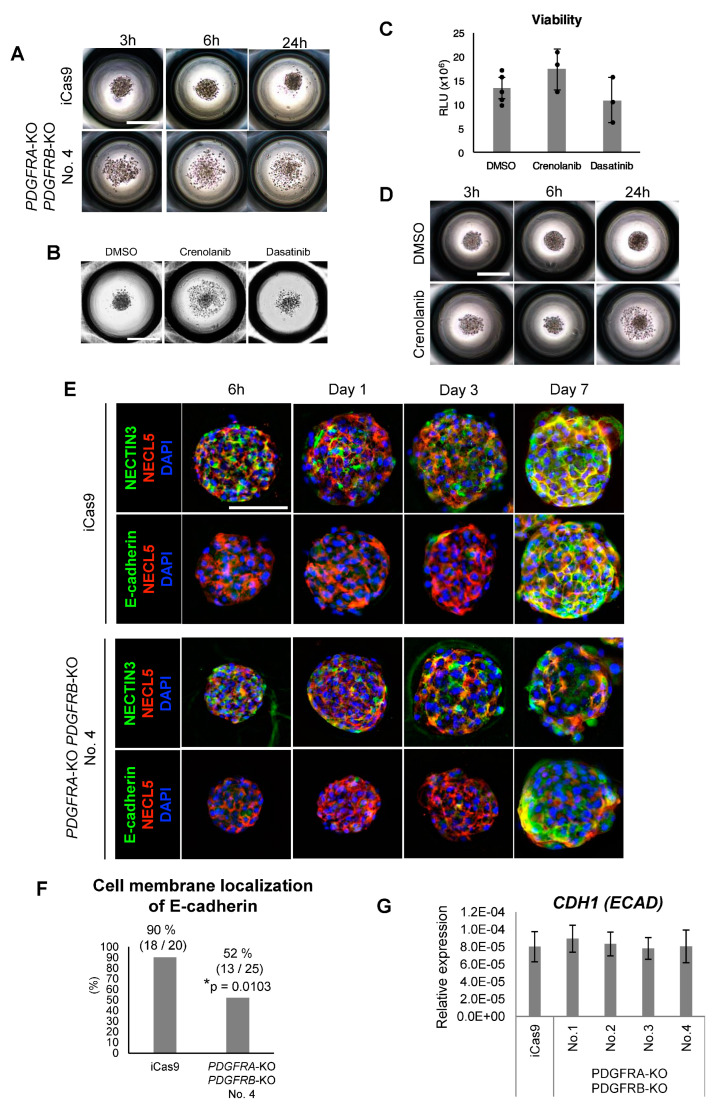
Relationship between PDGFRs and cell adhesion. (**A**) Morphologies of iCas9 and *PDGFRA/B* double KO No. 4 HS at 3, 6, and 24 h after production. Scale bar, 200 μm. (**B**) Morphologies of HS at 24 h after administration of Crenolanib and Dasatinib. Scale bar, 200 μm. (**C**) Cell viability of HS at 24 h after administration of Crenolanib and Dasatinib. The bars represent mean ± SD (n = 3 or 5). RLU: relative luminescent unit. (**D**) Morphologies of Crenolanib-administered HS at 3, 6, and 24 h after HS production. Scale bar, 200 μm. (**E**) Representative images of HS immunostained with E-cadherin, NECTIN3 (green), NECL5 (red). Nuclei were counterstained with DAPI (blue). Scale bar, 100 μm. (**F**) Percentages of HS with E-cadherin localized to the cell membrane in iCas9 (n = 20) and *PDGFRA/B* KO No. 4 (n = 25) cell lines. The *p*-value was calculated by Poisson distribution test. (**G**) mRNA levels of E-cadherin in day 7 HS of iCas9 and *PDGFRA/B* double KO cell lines. See also Figure S10.

Figure 3C shows the aggregation failure of HS in *PDGFRA* and *PDGFRB* single KO cell lines on day 1 after production. This result suggested that PDGF receptors may be related to cell aggregation and adhesion. Thus, we next attempted a detailed analysis of the relationship among PDGF receptors, cell aggregation, and cell adhesion. It was observed that *PDGFRA/B*-KO HS had already failed to aggregate at 3 h after HS production (Figure 6A). In addition, expression analysis of major cell adhesion molecules on d6DE day 10 cells showed little difference among iCas9 and *PDGFRA/B* double KO cell lines (Figure S10). These results suggested that PDGF receptors have a role in the very early stage of the cell aggregation and adhesion. To investigate the effect of PDGF signaling on the cell aggregation independent of PDGF signal activity before detachment from the 2D-culture dish, inhibitors of PDGF receptors were administered to d6DE day 10 cells derived from wild-type hiPSCs during HS production. Crenolanib (with an IC_50_ value of 10 nmol/L) is known to be most specific to PDGFRα/β in receptor tyrosine kinase inhibitors, and Dasatinib (with an IC_50_ value of 62 nmol/L) was used as a PDGFR inhibitor. It has been known that both Crenolanib and Dasatinib inhibit PDGF signaling by binding to the active site in the kinase domain of the receptors [17,18,19]. In the results, HS formation was hampered by both Crenolanib and Dasatinib on day 1 after production (Figure 6B). Their viabilities were at the same level as the mock control, which indicated that cell death is not induced (Figure 6C). Moreover, slight cell aggregation failure of HS was already observed at 3 h after Crenolanib treatment, although it was milder than the phenotype of *PDGFRA/B*-KO cell lines (Figure 6D). Therefore, it was indicated to be essential for HS formation that PDGF signaling is activated at the very early stage when cell adhesion is established shortly after the cells contact one another.

To analyze the effect of PDGF receptors and signaling on the localization of cell adhesion molecules in HS, we next performed immunostaining of cell adhesion molecules in HS of iCas9 and *PDGFRA/B* double KO cell lines (Figure 6E). Although some cases showed that *PDGFRA/B* double KO cell line displayed aggregation failure within 1 day after HS production, parts of slightly aggregated HS were used for immunostaining. Among cell adhesion molecules, it has been known that E-cadherin is essential for cell adhesion of epithelial cells; NECTIN3 and NECL5 establish adherens junctions such as E-cadherin, and both are the only ones in the nectin and nectin-like molecule family that can interact with PDGF receptors [20,21]. NECTIN3 and NECL5 had already localized on the cell membrane 6 h after HS production in both the iCas9 and *PDGFRA/B* double KO cell lines; their localization was maintained until day 3 (Figure 6E), whereas localization of E-cadherin to the cell membrane was not observed until day 3 (Figure 6E). These observations corresponded to the process of adherens junction formation by which intercellular binding of NECTINs or NECL5 initially occurs and then E-cadherin is subsequently recruited to the same cell adhesion site [22]. Unexpectedly, localizations of NECTIN3, NECL5, and E-cadherin were quite similar between iCas9 and *PDGFRA/B*-KO HS until day 3 (Figure 6E). However, on day 7 after HS production, NECTIN3, NECL5, and E-cadherin were localized on almost all cell membranes of iCas9 HS, whereas they were almost not observed on the cell membrane of *PDGFRA/B*-KO HS (Figure 6E). The rate of HS which had E-cadherin localized to their cell membranes was significantly decreased in *PDGFRA/B*-KO HS (Figure 6F). There was no difference among mRNA levels of E-cadherin of iCas9 and *PDGFRA/B*-KO HS on day 7 (Figure 6G), which implied that only recruitment of E-cadherin failed in *PDGFRA/B*-KO HS. In *PDGFRA/B* double KO cell lines, after aggregation failure on day 1, HS were visually reaggregated, and their viabilities were comparable with iCas9 control on day 11 (Figure 4B,C); however, these results demonstrated that the cell adhesion specific to epithelial cells is abnormal in *PDGFRA/B* double KO HS. The abnormality of cell adhesion might be related to the inhibition of HLC differentiation and maturation in *PDGFRA/B* double KO HS.

**Figure 7:**
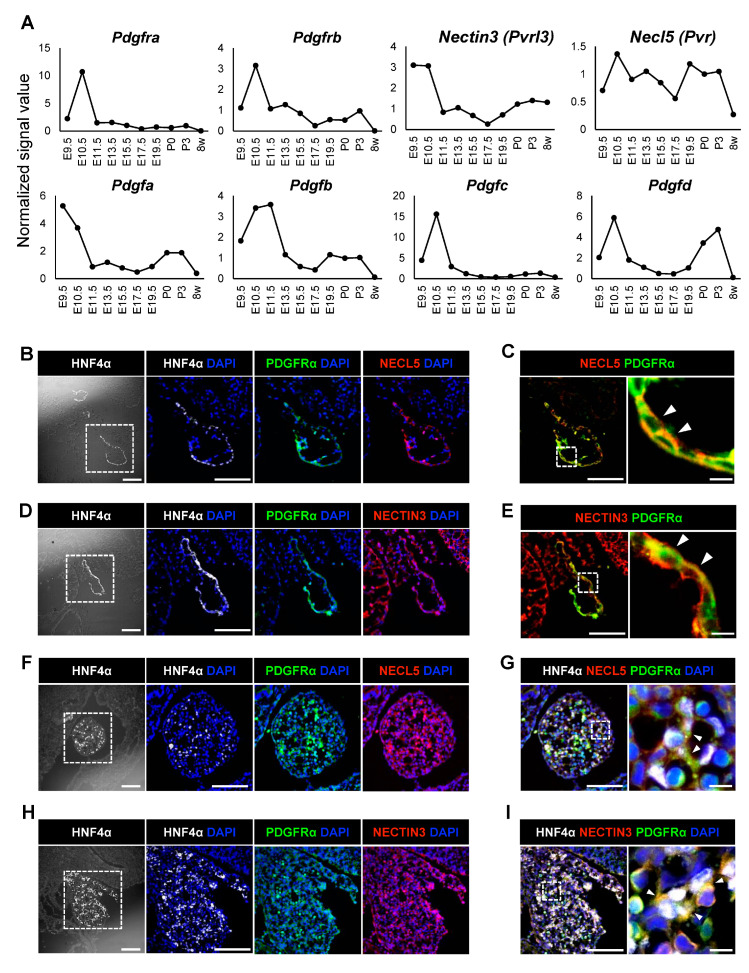
Relationship between PDGFR and in vivo mouse liver development. (**A**) mRNA levels of PDGFRs, PDGFs, NECTIN3, and NECL5 in mouse hepatoblasts or hepatocytes from early pregnancy to postnatal period. The signal values represent relative ratio to median across all samples. E: embryonic day, P: postnatal day, w: week old. (**B**–**I**) Immunohistochemical staining with HNF4α (white), PDGFRα (green), NECL5 and NECTIN3 (red). Nuclei were counterstained with DAPI (blue). (**B**–**E**) Immunostaining images whole-body sagittal sections of mouse E9.5 hepatoblasts. (**B**,**D**) The merged images with DAPI are enlargements of the dotted lines in the left panel. Scale bar, 100 μm. (**C**,**E**) Merged images of NECL5 or NECTIN3 and PDGFRα. The right panels are enlarged images of the dotted lines in the left panel. Scale bars of left and right panels are 100 μm and 10 μm, respectively. (**F**–**I**) Immunostaining images whole-body sagittal sections of mouse E10.5 hepatoblasts. (**F**,**H**) The merged images with DAPI are enlargements of the dotted lines in the left panel. Scale bar, 100 μm. (**G**,**I**) Merged images of NECL5 or NECTIN3, HNF4α, PDGFRα, and DAPI. The right panels are enlarged images of the dotted lines in the left panel. Scale bars of left and right panels are 100 μm and 10 μm, respectively. Arrowheads indicate co-localization of each protein.

Finally, we investigated the relationship between PDGFR and in vivo liver development because previous study has indicated that the HS of our protocol is similar to the human fetal liver in gestation week 10.5 to 17.5 by comprehensive gene expression analysis [23]. The expression levels of *Pdgfra*, *Pdgfrb*, *Pdgfa*, *Pdgfb*, *Pdgfc*, *Pdgfd*, *Nectin3*, and *Necl5* in hepatoblasts or hepatocytes extracted from mouse liver were evaluated from embryonic day (E) 9.5 to postnatal by using the microarray analysis data sets previously reported by our group [2]. Notably, all of these eight genes had the expression peak at or near E10.5 (Figure 7A). The expression patterns of *Pdgfra* and *Pdgfrb* were consistent with previous studies in which the protein levels of PDGFRα and PDGFRβ were transiently increased in mouse fetal livers of E10 to 11 [12,13]. *Pdgfra*, *Pdgfa*, *Pdgfb*, *Pdgfc*, and *Pdgfd* were strikingly up-regulated at or near E10.5 and then rapidly downregulated (Figure 7A), which was similar to the expression peaks of *PDGFRA*, *PDGFA*, *PDGFB*, *PDGFC*, and *PDGFD* during hiPSC-derived HLC differentiation (Figure 3B). These results imply that PDGF signaling is strongly activated in hepatoblasts at this developmental stage. In contrast, *Pdgfrb* also displayed the expression peak at E10.5, which was inconsistent with the *PDGFRB* expression pattern during hiPSC-derived HLC differentiation (Figure 3B and Figure 7A). These results might be caused by the difference between in vitro and in vivo or the interspecies difference. Because *Nectin3* and *Necl5* had the expression peak at E10.5 (Figure 7A), it can be assumed that they interact with PDGFRs and coordinately work to form intercellular adherens junctions.

Furthermore, localizations of PDGFRα, NECTIN3, and NECL5 at mouse E9.5 and E10.5 were analyzed by immunohistochemical staining. On whole-body sagittal sections of mouse fetuses at E9.5 and E10.5, cells with HNF4α-positive nuclei were found only in limited areas, which were considered hepatoblasts (Figure 7B,D–H). Surprisingly, at E9.5, the localization of PDGFRα and NECL5 were completely in agreement with hepatoblasts, although NECTIN3 expression was observed in both hepatoblasts and surrounding cells (Figure 7B–D). These results suggested that PDGFRα and NECL5 play specific roles in the very early stage of mouse fetal liver development. PDGFRα, NECTIN3, and NECL5 were co-localized in the cell membranes of hepatoblasts (Figure 7C–E), which implied that adherens junction was induced in hepatoblasts of E9.5, which have just begun to bud. In the E10.5 mouse fetus, HNF4α-positive hepatoblasts expanded and occupied a particular area compared to E9.5 (Figure 7F,H). PDGFRα, NECTIN3, and NECL5 were also co-localized on the cell membrane of almost all HNF4α-positive hepatoblasts at E10.5 (Figure 7F–I), which was consistent with the expression peak of *Pdgfra*, *Nectin3*, and *Necl5* at E10.5 (Figure 7A). These results suggested that PDGF receptors and signaling are involved in cell proliferation and cell adhesion when hepatoblasts rapidly proliferate from monolayer to form a 3D-structure, liver bud. Therefore, we presented that PDGF receptors and signaling have specific roles not only in vitro but also in vivo very early stages of liver development.

## 3. Discussion

When producing hiPSC-LO, 2D-cultured cells differentiated from hiPSCs must initially go through a simple 3D-aggregate, HS. However, little is known about the mechanism by which the 3D structure of HS is spontaneously formed from the 2D-cultured cells. This study aimed to identify factors that determine the character of the cells that have the potential for appropriate HS formation and maturation. Identifying the factors contributes to further understanding and developing 3D-culture systems of HLC or associated cells.

In this study, we present that *PDGFRA* is specifically induced in the cells suitable for HS formation and demonstrate the requirement of PDGF receptors and signaling for HS formation and maturation. Additionally, in vivo, we show that the localization of PDGFRA and NECL5 are completely in agreement with E9.5 hepatoblasts, which begin to form 3D-structural liver buds from a single layer.

PDGF receptors have been well known as the marker of MC. However, our results newly suggest that *PDGFRA* is also the marker gene indicating the potential for HS formation and maturation in the HLC differentiation process. Among researchers of HS or LO, it had empirically been known that only specific cells in the HLC differentiation process could form appropriate HS [2,6]. We propose that *PDGFRA* expression level may be available for selecting cells suitable for HS production.

Hepatocyte growth factor, oncostatin M, and dexamethasone have been well known as the molecules promoting maturation of HLCs differentiated from pluripotent stem cells [24,25,26]. This study adds novel players, PDGFRs and PDGFs, to these molecules. This PDGF signaling effect on HLC was found only in the 3D-culture system and did not emerge in the conventional 2D-culture system, which may have delayed the discovery of its effect. We expect that further studies on the relationship between PDGF signaling and HLC maturation can reveal the mechanism of hepatocyte differentiation.

Our results suggest that PDGF receptors play roles in initial cell aggregation of HS. However, we could not wholly reveal the mechanism between initial cell aggregation of HS and PDGF receptors. Because expression levels of almost cell adhesion molecules predicted to work in epithelial cells were comparable among iCas9 and *PDGFRA/B* double KO cell lines, it can be speculated that PDGFRs directly contribute to cell adhesion. Indeed, it has been reported that PDGFRs coordinate with NECTINs and NECL5 to be involved in the formation of adherens junctions [22]. Accordingly, future studies on the involvement of PDGFRs in cell adhesion may elucidate the HS’s initial cell aggregation mechanism.

It was suggested that PDGFRα and NECL5 play specific roles in the very early stage of mouse fetal liver development. Notably, it has been reported that expression of *Necl5* is deficient in almost all adult mouse organs whereas substantially induced in regenerating liver [27]. Furthermore, it has also been shown that NECL5 enhances PDGF-induced activation of the Ras–Raf–MEK–ERK pathway and PDGF-induced cell proliferation by shortening the period of the G (0) / G (1) phase of the cell cycle [28,29]. These observations suggest that PDGFRα and NECL5 may be closely involved in rapid proliferation and associated cell adhesion in E9.5 hepatoblasts that begin forming 3D-structural liver buds from a single layer.

In mouse liver development, mRNA levels of PDGFRs and PDGFs in hepatoblasts or hepatocytes were decreased after E10.5. Because hepatocyte differentiation also progresses after E10.5 in mouse liver development, it is unclear whether in vivo hepatocyte differentiation is promoted by PDGF signaling in an autocrine manner. However, the previous microarray study showed that expression levels of *PDGFA* and *PDGFB* gradually increase from E11.5 to E18.5 in mouse placenta [30]. Because the liver receives blood inflow from the placenta first among all organs, it seems highly probable that the liver is directly exposed to PDGFs derived from the placenta after E11.5. Therefore, it can be assumed that PDGF signaling also affects in vivo liver development.

Notably, our results suggest that PDGFRs and PDGFs may relate to the 3D-structuring of hepatocyte progenitors in vitro and in vivo. However, many unclear issues remain about how PDGFRs and PDGFs function in vivo liver development. In a previous study, it has been shown in vitro that PDGFRα contributes to the proliferation of hepatoblasts isolated from E10 mouse fetal liver, whereas hepatocyte-specific conditional *Pdgfra* KO (*Pdgfra*^loxp/loxp^; *Alb-Cre*) mice have not displayed any apparent phenotypes in liver development [13]. However, the localization of PDGFRα was entirely in agreement with HNF4A-positive hepatoblasts in E9.5 fetuses, which reminds us that HNF4A-positive hepatoblast-specific conditional *Pdgfra* KO (*Pdgfra*^loxp/loxp^; *Hnf4A-Cre*) mice may show phenotypes in liver development. Future studies are expected to provide clues for elucidating relationships among PDGF receptors, signaling, and in vivo liver development.

## 4. Materials and Methods

### 4.1. iPSC Maintenance

The human iPSC line Ff-I01s04 was provided by Kyoto University. Accession Number of Ff-I01s04 is ‘CVCL_C1DY’ in Cellosaurus of Expasy (Swiss Bioinfomatics Resource Portal). As we previously reported [14], all iPSC lines were maintained using the dish coated by iMatrix-511 silk (Nippi, Tokyo, Japan) and StemFit AK02N medium (ReproCELL, Yokohama, Japan). The medium was changed at day 1, 3, 5, and 6; the detachment for passage and differentiation was performed using accutase (Innovative Cell Technologies, San Diego, CA, USA) at day 7. The use of human iPSCs was approved by the ethics committee of The Institute of Medical Science, The University of Tokyo (Approval Code: 2019-4-0716).

### 4.2. Hepatocyte-Like Cells Differentiation

The cells were differentiated as described previously [14]. Briefly, iPSCs were seeded into differentiation medium supplemented with Rho-associated coiled-coil forming kinase (ROCK) inhibitor Y-27632 (Fujifilm Wako Pure Chemical, Osaka, Japan) on dishes coated with the iMatrix-511 silk. The first differentiation medium for DE was produced using RPMI 1640 (Fujifilm Wako Pure Chemical, Japan) as base medium and supplemented with 2% B-27 supplement (Thermo Fisher Scientific, Waltham, MA, USA), 100 ng/mL Activin A (Ajinomoto, Tokyo, Japan) (day 0 to 6), CHIR99021 (2 μM; Cayman Chemical, Ann Arbor, MI, USA) (day 0 to 3), and 0.5 mM sodium butylate (Sigma Aldrich, MO, USA) (day 1 to 4). In d4DE protocol, the day 4 cells were defined as DE; in d6DE protocol, the day 6 cells were defined as DE.

In next differentiation step, StemFit Basic02N (Ajinomoto, Japan), which we previously developed [14], was adopted for HE and IH differentiation. 1% dimethyl sulfoxide (DMSO) (Nacalai Tesque, Kyoto, Japan), 0.1 mM 2-mercaptoethanol (2-ME), 0.5% L-glutamine, and 1% non-essential amino acids (NEAA) (Thermo Fisher Scientific, MA, USA) were added to StemFit Basic02N, which was used for medium change every day until day 12.

The last step of differentiation to induce MH was slightly modified from our previous protocol [14]. 5% FBS (BioWest, Nuaillé, France), 100 nM dexamethasone (DEX) (Sigma Aldrich, St. Louis, MO, USA), and 20 ng/mL oncostatin M (OSM) (R&D Systems, Minneapolis, MN, USA) were added to D-MEM (Fujifilm Wako Pure Chemical, Japan), which was used for medium change at day 13, 17, and 20.

### 4.3. Mesenchymal Cells Differentiation

MCs were differentiated as described previously [2]. Briefly, human iPSCs were plated on dishes coated with the iMatrix-511 silk and cultured in StemFit AK02N for 2 days. The 10 μM ROCK inhibitor Y-27632 was used only on the first day. Then, the medium was replaced with mesoderm induction medium composed of D-MEM/F12 (Thermo Fisher Scientific, MA, USA) with 1% Glutamax (Thermo Fisher Scientific, MA, USA) and 1% B27 with 8 μM CHIR99021 and 25 ng/mL BMP4 (Fujifilm Wako Pure Chemical, Japan), followed by 3-day exposure of 2 ng/mL activin A and 10 ng/mL PDGFBB (R&D Systems, MN, USA). After 3 days, the mesoderm induction medium was replaced with MC induction medium consistent with StmePro-34 SFM (Thermo Fisher Scientific, MA, USA) supplemented with 10 ng/mL FGF2 (Fujifilm Wako Pure Chemical, Japan) and 10ng/mL PDGFBB for 3 days.

### 4.4. Hepatic Spheroids Production

The HS were produced as previously described [14]. Briefly, the 2D-cultured cells during HLC differentiation were dissociated with 0.05 % Trypsin-EDTA (Thermo Fisher Scientific, MA, USA) and inoculated into Elplasia 24-well plate (Corning, Corning, NY, USA). The 10 μM ROCK inhibitor Y-27632 was used only on the first day. The HS were cultured in D-MEM: KBM VEC-1 (KOHJIN BIO, Saitama, Kyoto, Japan) (1:1) supplemented with 2.5 % FBS, 50 nM DEX, and 10 ng/mL OSM. Half of the medium was replaced daily. For PDGFs treatment experiments, PDGF-AA, -AB, -BB, and -CC were used at 100 ng/mL. For PDGFR inhibitor experiments, Crenolanib and Dasatinib were used at 2.5 nM and 5.0 μM, respectively. The area of HS images was evaluated using a Cell3iMager duos (SCREEN Holdings, Kyoto, Japan).

### 4.5. Construction of Knockout Cell Line

Each gene-knockout iPSC line was produced by the method based on iCRISPR, a genome-engineering platform in hPSCs developed by González et al. [15]. Briefly, a plasmid Puro-iCas9 donor harboring doxycycline (DOX)-driven Cas9 sequences was introduced into Ff-I01s04, which was designated the iCas9 cell line in this paper. At first, 2 μg/mL DOX was treated from the day before passage to the second day after passage (total 3 days). For gRNAs, Alt-R CRISPR-Cas9 crRNA and Alt-R CRISPR-Cas9 tracrRNA synthesized by Integrated DNA Technologies (Coralville, IA, USA) were annealed according to the manufacturer’s manual. The Alt-R CRISPR-Cas9 crRNA sequences for each target gene were designed on the website developed by Concordet et al. [31], and those with the top three specificity scores that were considered unlikely to cause off-target effects were selected. The MIT specificity score and cutting frequency determination (CFD) specificity score were used as specificity scores [32,33], and both were confirmed to be higher than 90 for all of the designed crRNA sequences. The gRNAs were transfected using viafect (Promega, Fitchburg, WI, USA) during passage. The gRNA transfection was performed again on the next day after the passage. These cells were again passaged at low density (300 to 500 cells/cm^2^) for picking up single colonies 3–4 days after seeding. Single colonies were picked up aseptically under a microscope, and those derived from single colonies were used as individual cell lines. DNA sequencing analysis for knockout confirmation was requested from eurofins genomics, and Sanger sequencing was performed.

### 4.6. Flow Cytometry

Cells were labeled with APC anti-CD184 (CXCR4) antibody (BD Biosciences, Franklin Lakes, NJ, USA), Alexa Fluor 647 anti-CD140a (PDGFRα) antibody (BD Pharmingen, Franklin Lakes, NJ, USA), and PE anti-CD140b (PDGFRβ) antibody (BD Biosciences, NJ, USA). The analyses were performed by BD FACSCelesta Flow Cytometer (BD Biosciences, NJ, USA). The data were analyzed using BD FlowJo v10.8 software (BD Biosciences, NJ, USA).

### 4.7. Immunostaining

The immunostaining was performed as previously described [14]. See Table S2 for a list of primary antibodies. Alexa fluor-conjugated antibodies (Thermo Fisher Scientific) were used as secondary antibodies. DAPI (nacalai tesque) was used for staining cell nuclei. HS was stabilized on a slide glass using a Smear Gell (GenoStaff, Tokyo, Japan) according to the manufacturer’s protocol. Frozen blocks of mouse E9.5 and E10.5 fetuses were sectioned at 10 μm, and they were pasted on the slide glass. The images after immunostaining were taken using THUNDER Imaging Systems (Leica Microsystems, Wetzlar, Germany).

### 4.8. qRT-PCR

RNA was extracted using the PureLink RNA Mini Kit (Thermo Fisher Scientific, MA, USA). cDNA was synthesized using the High-Capacity cDNA Reverse Transcription Kit (Thermo Fisher Scientific, MA, USA). The quantitative PCR was performed using the THUNDERBIRD Probe qPCR Mix (TOYOBO, Osaka, Japan) and the Universal ProbeLibrary System (Roche Molecular Systems, Pleasanton, CA, USA). All probes and primers used for qPCR are presented in Table S3. 18S rRNA expression levels were quantified using Eukaryotic 18S rRNA Endogenous Control (Thermo Fisher Scientific, MA, USA).

### 4.9. Cell Viability Assay

Each cell suspension was prepared in 50 μL PBS and transferred to 96-well plate. The cell viabilities were measured using CellTiter-Glo Luminescent Cell Viability Assay (Promega, WI, USA) and GloMax Discover Microplate Reader (Promega, WI, USA).

### 4.10. ALB ELISA

The samples were collected from the culture medium at 24 h after medium change. Human Albumin ELISA Kit (ab179887) (abcam, Pleasanton, UK) was used according to the manufacturer’s instructions. ALB secretion levels were normalized by the cell viability value.

### 4.11. Quantification of Urea Production

The quantification of urea production was performed as previously described [14]. Briefly, the HS or MH were loaded with 2 mM NH_4_Cl in 2 % B27/RPMI and were incubated for 24 h. The medium was collected and measured by the QuantiChromTM urea assay kit (BioAssay Systems, Hayward, CA, USA), according to the manufacturer’s instructions. Urea production levels were normalized by the cell viability value.

### 4.12. Microarray Analysis

RNA samples were hybridized on SurePrint G3 Human GE v2 8×60K Microarray (Agilent Technologies, Santa Clara, CA, USA) according to the manufacturer’s instructions. To detect the differentially expressed genes, the normalization and comparison analyses were performed using R and *limma* package [34].

### 4.13. Statistical Analyses

Data were expressed as means ± standard deviation of independent experiments. All experiments were performed with at least three different cell preparations. The statistical significance was assessed by Student’s t test for gene expression analyses, unless otherwise specified. One-way analysis of variance (ANOVA) with post-hoc Tukey honestly significant difference (HSD) test was employed for analyzing data presented in Figure 2B,C; Poisson distribution test was employed in Figure 6F. The *p* values of < 0.05 were considered statistically significant.

### 4.14. Data and Code Availability

The microarray data have been deposited in Gene Expression Omnibus, the accession number is GSE215379.

## Data Availability

All raw data are available upon request.

## Supplementary Materials

The supporting information can be downloaded at: https://www.mdpi.com/article/10.3390/ijms24087075/s1.

## References

[1] Takebe T., Sekine K., Enomura M., Koike H., Kimura M., Ogaeri T., Zhang R.R., Ueno Y., Zheng Y.W., Koike N. (2013). Vascularized and functional human liver from an iPSC-derived organ bud transplant. Nature.

[2] Takebe T., Sekine K., Kimura M., Yoshizawa E., Ayano S., Koido M., Funayama S., Nakanishi N., Hisai T., Kobayashi T. (2017). Massive and reproducible production of liver buds entirely from human pluripotent stem cells. Cell Rep..

[3] Wu F., Wu D., Ren Y., Huang Y., Feng B., Zhao N., Zhang T., Chen X., Chen S., Xu A. (2019). Generation of hepatobiliary organoids from human induced pluripotent stem cells. J. Hepatol..

[4] Ouchi R., Togo S., Kimura M., Shinozawa T., Koido M., Koike H., Thompson W.L., Karns R., Mayhew C., McGrath P. (2019). Modeling Steatohepatitis in Humans with Pluripotent Stem Cell-Derived Organoids. Cell Metab..

[5] Ramli M.N.B., Lim Y.S., Koe C.T., Demircioğlu D., Tng W.Q., Andrew K., Gonzales U., Tan C.P., Szczerbinska I., Liang H. (2020). Human Pluripotent Stem Cell-Derived Organoids as Models of Liver Disease. Gastroenterology.

[6] Kulkeaw K., Tubsuwan A., Tongkrajang N., Whangviboonkij N. (2020). Generation of human liver organoids from pluripotent stem cell-derived hepatic endoderms. PeerJ.

[7] Harrison S.P., Baumgarten S., Verma R., Lunov O., Dejneka A., Sullivan G. (2021). Liver Organoids: Recent Developments, Limitations and Potential. Front. Med..

[8] Hoch R., Soriano P. (2003). Roles of PDGF in animal development. Development.

[9] Andrae J., Gallini R., Betsholtz C. (2008). Role of platelet-derived growth factors in physiology and medicine. Genes Dev..

[10] Demoulin J., Essaghir A. (2014). PDGF receptor signaling networks in normal and cancer cells. Cytokine Growth Factor Rev..

[11] Guérit E.M., Arts F., Dachy G., Boulouadnine B., Demoulin J. (2021). PDGF receptor mutations in human diseases. Cell Mol. Life Sci..

[12] Stock P., Monga D., Tan X., Micsenyi A., Loizos N., Monga S. (2007). Platelet-derived growth factor receptor-α: A novel therapeutic target in human hepatocellular cancer. Mol. Cancer Ther..

[13] Awuah P., Nejak-Bowen K., Monga S. (2013). Role and regulation of PDGFRα signaling in liver development and regeneration. Am. J. Pathol..

[14] Sekine K., Ogawa S., Tsuzuki S., Kobayashi T., Ikeda K., Nakanishi N., Takeuchi K., Kanai E., Otake Y., Okamoto S. (2020). Generation of human induced pluripotent stem cell-derived liver buds with chemically defined and animal origin-free media. Sci. Rep..

[15] González F., Zhu Z., Shi Z., Lelli K.M., Verma N., Li Q.V., Huangfu D. (2014). An iCRISPR platform for rapid, multiplexable, and inducible genome editing in human pluripotent stem cells. Cell Stem Cell.

[16] Zhu Z., Gonzãlez F., Huangfu D. (2014). The iCRISPR platform for rapid genome editing in human pluripotent stem cells. Methods Enzymol..

[17] Wu P., Nielsen T.E., Clausen M.H. (2015). FDA-approved small-molecule kinase inhibitors. Trends Pharmacol. Sci..

[18] Roskoski R. (2018). The role of small molecule platelet-derived growth factor receptor (PDGFR) inhibitors in the treatment of neoplastic disorders. Pharmacol. Res..

[19] Klug L.R., Kent J.D., Heinrich M.C. (2018). Structural and clinical consequences of activation loop mutations in class III receptor tyrosine kinases. Pharmacol. Ther..

[20] Kanzaki N., Ogita H., Komura H., Ozaki M., Sakamoto Y., Majima T., Ijuin T., Takenawa T., Takai Y. (2008). Involvement of the nectin-afadin complex in PDGF-induced cell survival. J. Cell Sci..

[21] Amano H., Ikeda W., Kawano S., Kajita M., Tamaru Y., Inoue N., Minami Y., Yamada A., Takai Y. (2008). Interaction and localization of Necl-5 and PDGF receptor β at the leading edges of moving NIH3T3 cells: Implications for directional cell movement. Genes Cells.

[22] Takai Y., Miyoshi J., Ikeda W., Ogita H. (2008). Nectins and nectin-like molecules: Roles in contact inhibition of cell movement and proliferation. Nat. Rev. Mol. Cell Biol..

[23] Camp J.G., Sekine K., Gerber T., Loeffler-Wirth H., Binder H., Gac M., Kanton S., Kageyama J., Damm G., Seehofer D. (2017). Multilineage communication regulates human liver bud development from pluripotency. Nature.

[24] Si-Tayeb K., Noto F.K., Nagaoka M., Li J., Battle M.A., Duris C., North P.E., Dalton S., Duncan S.A. (2010). Highly efficient generation of human hepatocyte–like cells from induced pluripotent stem cells. Hepatology.

[25] Zhao D., Chen S., Duo S., Xiang C., Jia J., Guo M., Lai W., Lu S., Deng H. (2013). Promotion of the efficient metabolic maturation of human pluripotent stem cell-derived hepatocytes by correcting specification defects. Cell Res..

[26] Ang L.T., Tan A.K., Autio M.I., Goh S.H., Choo S.H., Lee K.L., Tan J., Pan B., Lee J.J., Lum J.J. (2018). A Roadmap for Human Liver Differentiation from Pluripotent Stem Cells. Cell Rep..

[27] Erickson B.M., Thompson N.L., Hixson D.C. (2006). Tightly regulated induction of the adhesion molecule necl-5/CD155 during rat liver regeneration and acute liver injury. Hepatology.

[28] Kakunaga S., Ikeda W., Shingai T., Fujito T., Yamada A., Minami Y., Imai T., Takai Y. (2004). Enhancement of Serum- and Platelet-derived Growth Factor-induced Cell Proliferation by Necl-5/Tage4/Poliovirus Receptor/CD155 through the Ras-Raf-MEK-ERK Signaling. J. Biol. Chem..

[29] Hirota T., Irie K., Okamoto R., Ikeda W., Takai Y. (2005). Transcriptional activation of the mouse Necl-5/Tage4/PVR/CD155 gene by fibroblast growth factor or oncogenic Ras through the Raf–MEK–ERK–AP-1 pathway. Oncogene.

[30] Soncin F., Khater M., To C., Pizzo D., Farah O., Wakeland A., Arul Nambi Rajan K., Nelson K.K., Chang C., Moretto-Zita M. (2018). Comparative analysis of mouse and human placentae across gestation reveals species-specific regulators of placental development. Development.

[31] Concordet J., Haeussler M. (2018). CRISPOR: Intuitive guide selection for CRISPR/Cas9 genome editing experiments and screens. Nucleic Acids Res..

[32] Doench J.G., Fusi N., Sullender M., Hegde M., Vaimberg E.W., Donovan K.F., Smith I., Tothova Z., Wilen C., Orchard R. (2016). Optimized sgRNA design to maximize activity and minimize off-target effects of CRISPR–Cas9. Nat. Biotechnol..

[33] Tycko J., Wainberg M., Marinov G.K., Ursu O., Hess G.T., Ego B.K., Aradhana, Li A., Truong A., Trevino A.E. (2019). Mitigation of off-target toxicity in CRISPR-Cas9 screens for essential non-coding elements. Nat. Commun..

[34] Ritchie M.E., Phipson B., Wu D., Hu Y., Law C.W., Shi W., Smyth G.K. (2015). *limma* powers differential expression analyses for RNA-sequencing and microarray studies. Nucleic Acids Res..

