# Supplementary material for: PDGF Receptors and Signaling Are Required for 3D-Structure Formation and Differentiation of Human iPSC-Derived Hepatic Spheroids
Source: Int J Mol Sci. 2023 Apr 11;24(8):7075. doi: 10.3390/ijms24087075 (PMC10138705; doi:10.3390/ijms24087075)
Supplement: Supplementary file 1 [file ijms-24-07075-s001.zip › 230317_Supplemental_Figure_Table/230315_FigureS_TableS.pdf]

Figure S1

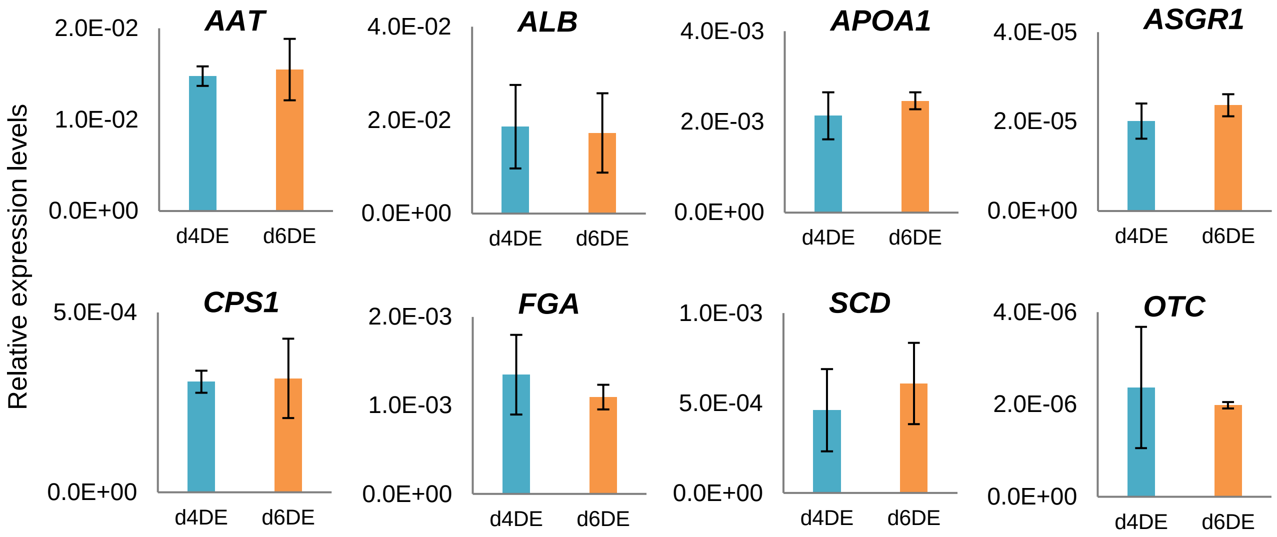

Figure S1. Gene expression analysis in d4DE and d6DE MH, related to Figure 1.
Normalization is performed using the expression level of 18S rRNA.
Data are represented as mean ± SD. (n = 4 to 6).

Figure S2

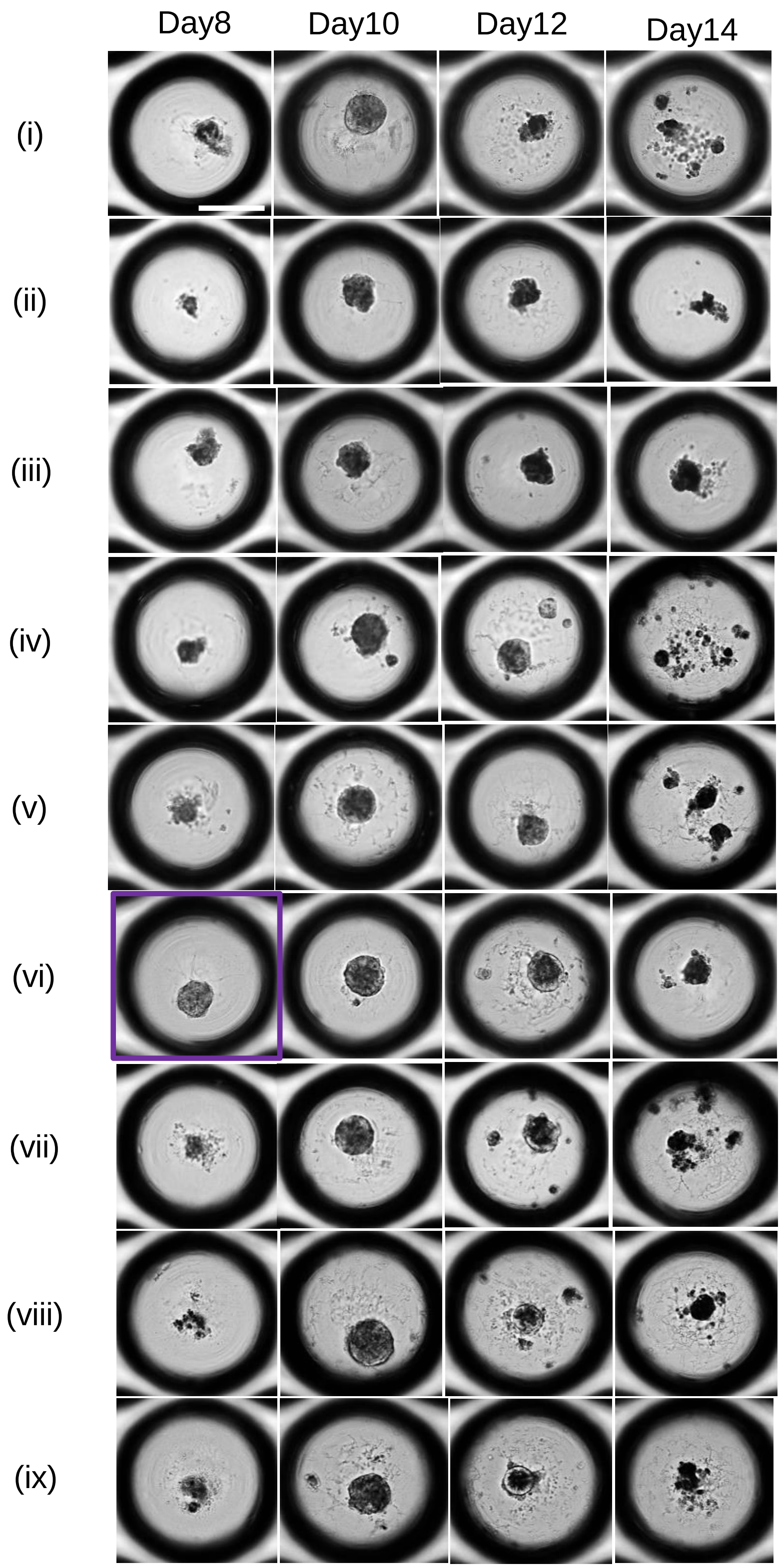

Figure S2. Repeatedly produced HS, related to Figure 2.
Morphologies of nine batches of HS produced on days 8, 10, 12 and 14 in the d6DE protocol ((i) to (ix)). HS produced from exceptional d6DE day 8 (Ex-d6DE day 8) cells are outlined in purple (vi). Scale bar, 200 μm.

Figure S3

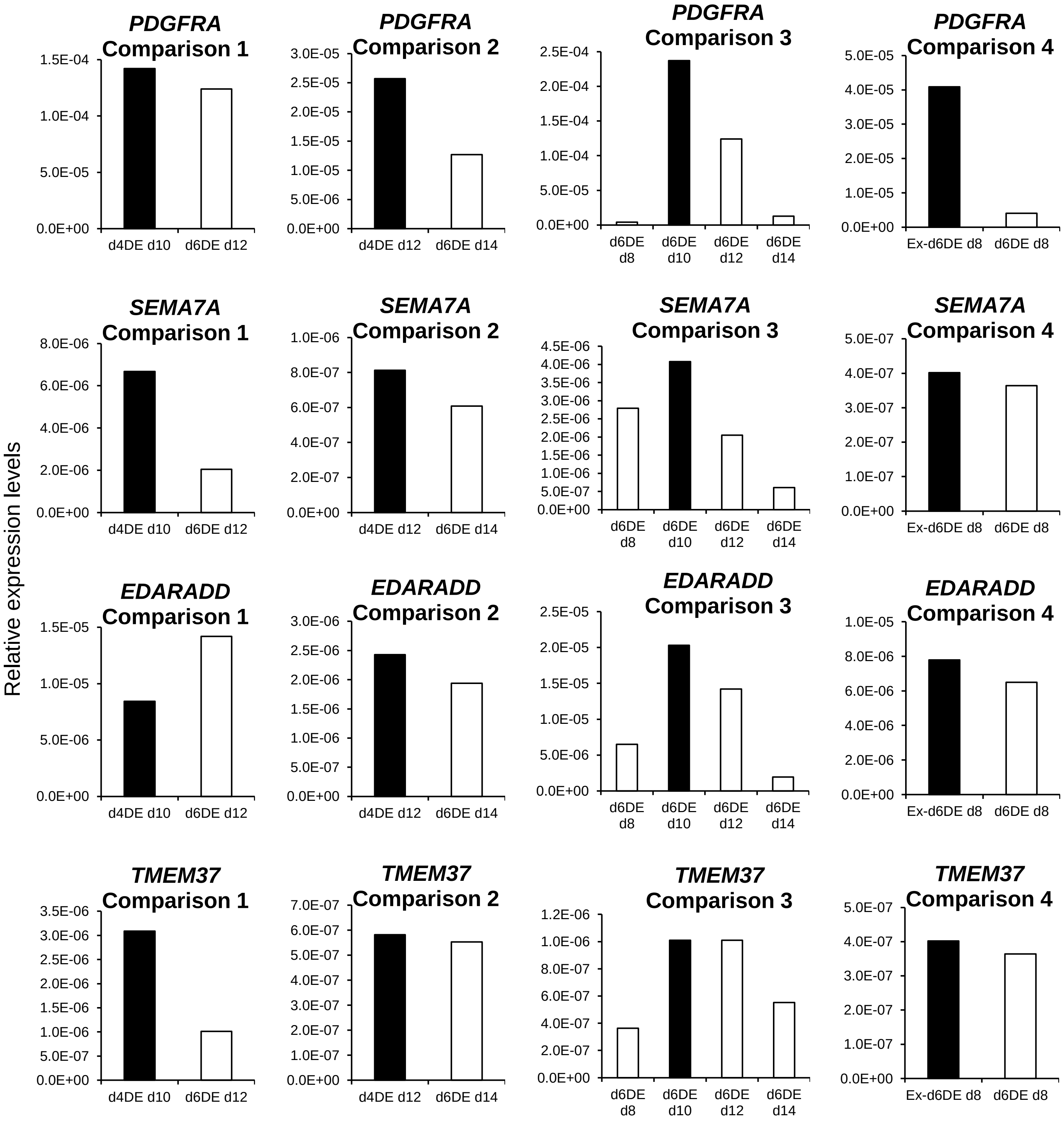

Figure S3. Verification of microarray analysis by qRT-PCR, related to Figure 2.
The black and white bars represent the cells with better and worse HS-forming ability, respectively. Normalization is performed using the expression level of 18S rRNA.

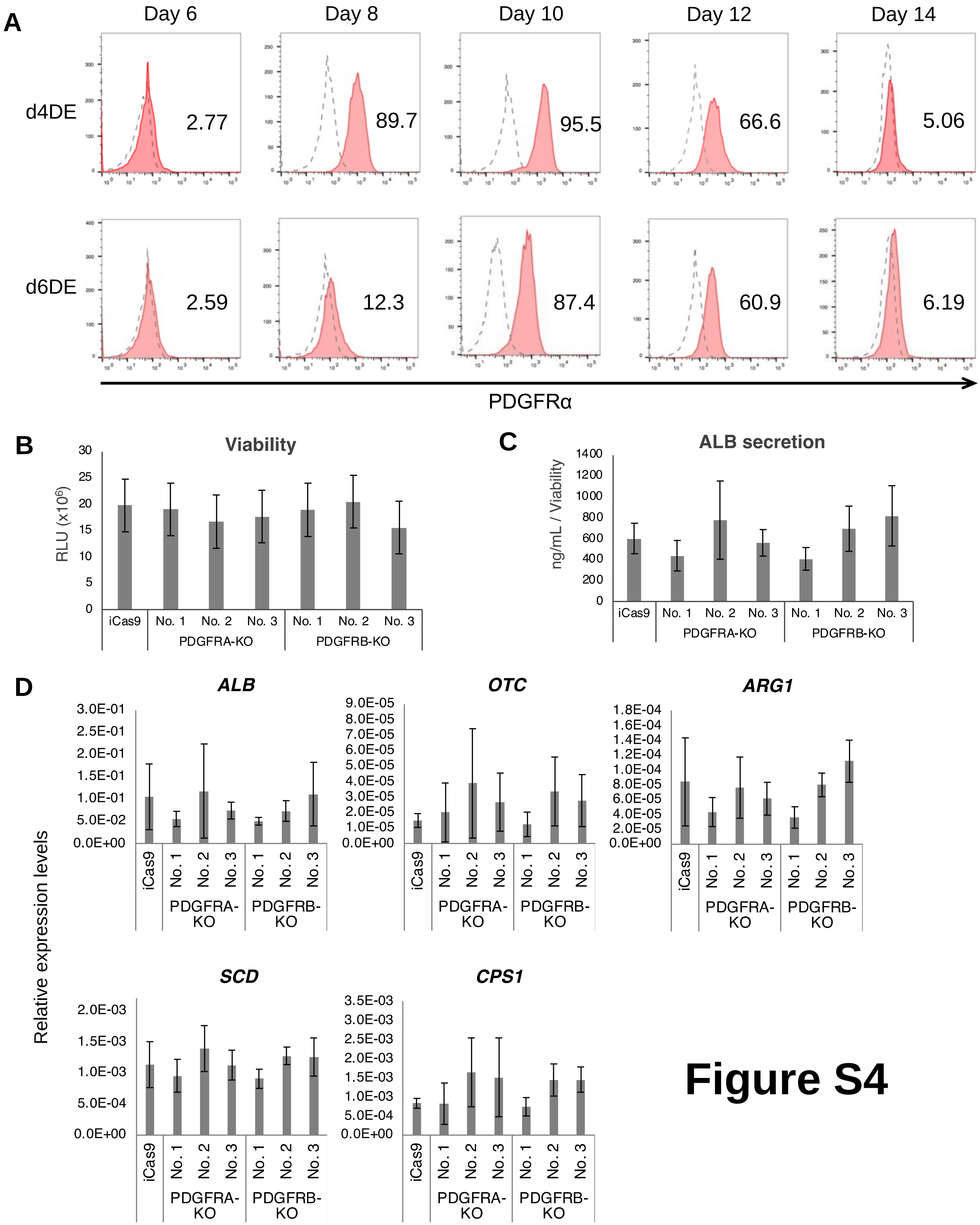

Figure S4. Functional analyses of PDGFRs in differentiating cells and HS, related to Figure 3.
(A) Flow cytometric analysis of PDGFRα. The dotted lines are the isotype control, the red lines are the sample stained with PDGFRα Alexa Fluor 647-conjugated antibody, and the numbers represent the positive rate (%).
(B) Cell viability within HS by measuring intracellular ATP levels in iCas9, *PDGFR*-KO, and *PDGFRB*-KO cell lines. The bars represent mean ± SD. (n = 3). RLU: relative luminescent unit.
(C) Albumin secretion of each HS on day 11 after HS production in iCas9, *PDGFR*-KO, and *PDGFRB*-KO cell lines. The bars represent mean ± SD. (n = 3).
(D) Expression levels of hepatocyte marker genes in iCas9, *PDGFR*-KO, and *PDGFRB*-KO cell lines. Normalization is performed using the expression level of 18S rRNA. The bars represent mean ± SD. (n = 4).

Figure S5

A

iCas9: GGACTTCCCATCCGGCGTTCCTGGTCTTAGGCTGT
PDGFRA-KO No. 1: GGACTTCCCATCTTAAGACCGGCGTTCCTGGTCTTAGGCTGT 7 bases insertion
PDGFRA-KO No. 2: GGACTTCCCATC--------------TTAGGCTGT 14 bases deletion
PDGFRA-KO No. 3: GGACTTCCCATC-------------CTTAGGCTGT 13 bases deletion

B

iCas9:
CCTTCGTTCTGACCTGCTCGGGTTCAGCTCCGGTGGTGTGGGAACGGATGTCCCAGGAGCCCCCACAGGAAATGGC
CAAGGCCCAGGATGGCACCTTCTCCAGCGTGCTCACACTGACCAACCTCACTGGGCTAGACACGGGAGAATACTTT
TGCACCCACAATGACTCCCGTGGACTGGAGACCGATGAGCGGAAACGGCTCTAC

PDGFRB-KO No. 1: CCTTCG---(183 bases deletion)---AGCGGAAACGGCTCTAC
PDGFRB-KO No. 2: CCTTCG---(183 bases deletion)---AGCGGAAACGGCTCTAC
PDGFRB-KO No. 3: CCTTCG---(183 bases deletion)---AGCGGAAACGGCTCTAC

C

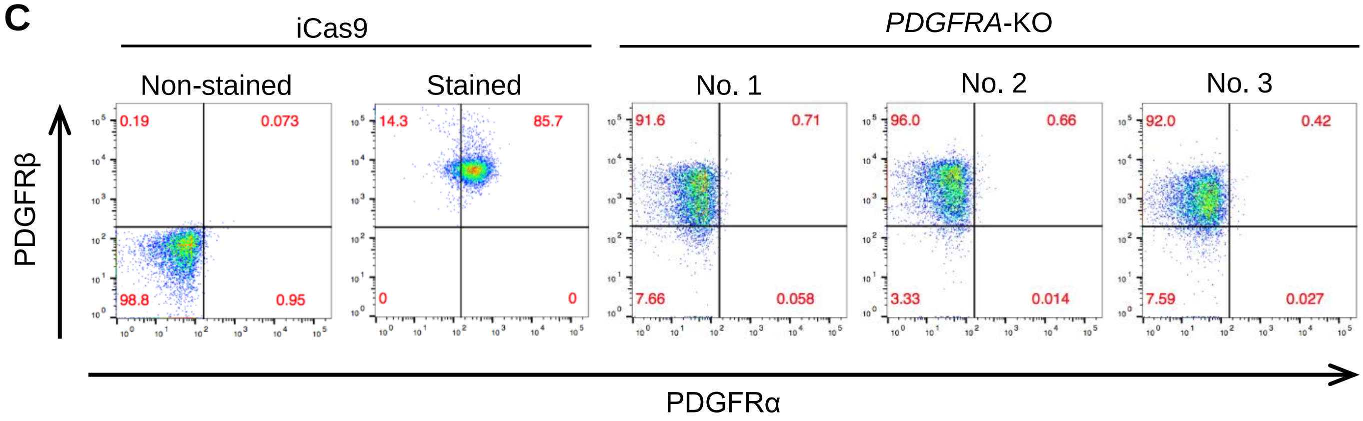

D

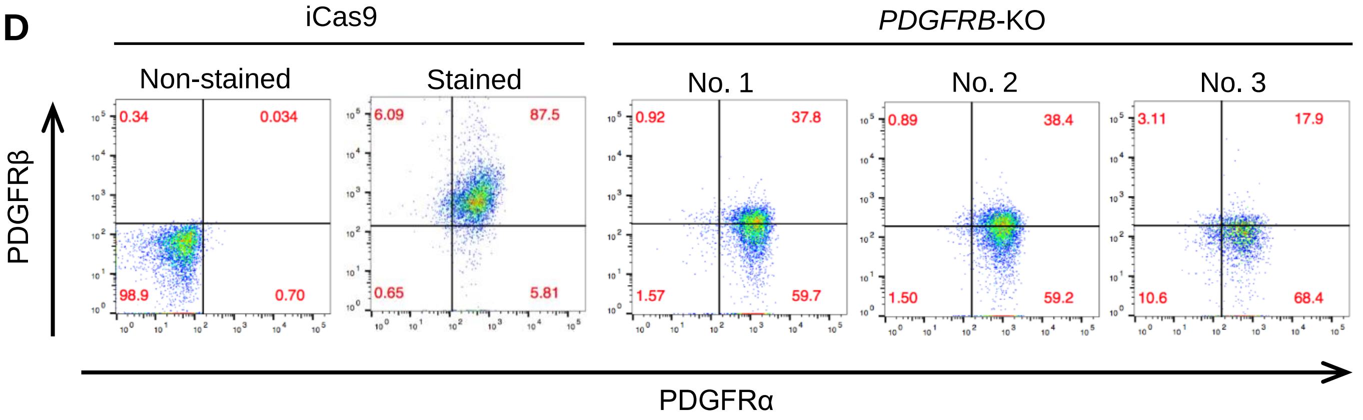

Figure S5. Validation of *PDGFRA*-KO and *PDGFRB*-KO cell lines, related to Figure 3.
(A) Sequence analysis of *PDGFRA*-KO cell lines. The yellow bases represent a target sequence, the underlined bases are protospacer adjacent motif in CRISPR/Cas9 system.
(B) Sequence analysis of *PDGFRB*-KO cell lines. The cyan and magenta bases represent target sequences, the underlined bases are protospacer adjacent motifs in CRISPR/Cas9 system.
(C) (D) Flow cytometric analysis of PDGFRα and PDGFRβ in *PDGFRA*-KO and *PDGFRB*-KO cell lines. Each cell line was differentiated into MC using PDGF-BB (C) or PDGF-AA (D) and then analyzed. The numbers represent the population rates (%).

Figure S6

A

```
PDGFRB-KO No. 1 :    GGACTTCCCATCCGGCGTTCCTGGTCTTAGGCTGT
PDGFRA/B-KO No. 1:   GGACTTCCCATC----------GGTCTTAGGCTGT    10 bases deletion
PDGFRA/B-KO No. 2:   GGACTTCCCATC-------------CTTAGGCTGT    13 bases deletion
PDGFRA/B-KO No. 3:   GGACTTCCCATC-----------------GGCTGT    17 bases deletion
PDGFRA/B-KO No. 4:   GGACTTCCCATC-----------GTCTTAGGCTGT    11 bases deletion
```

B

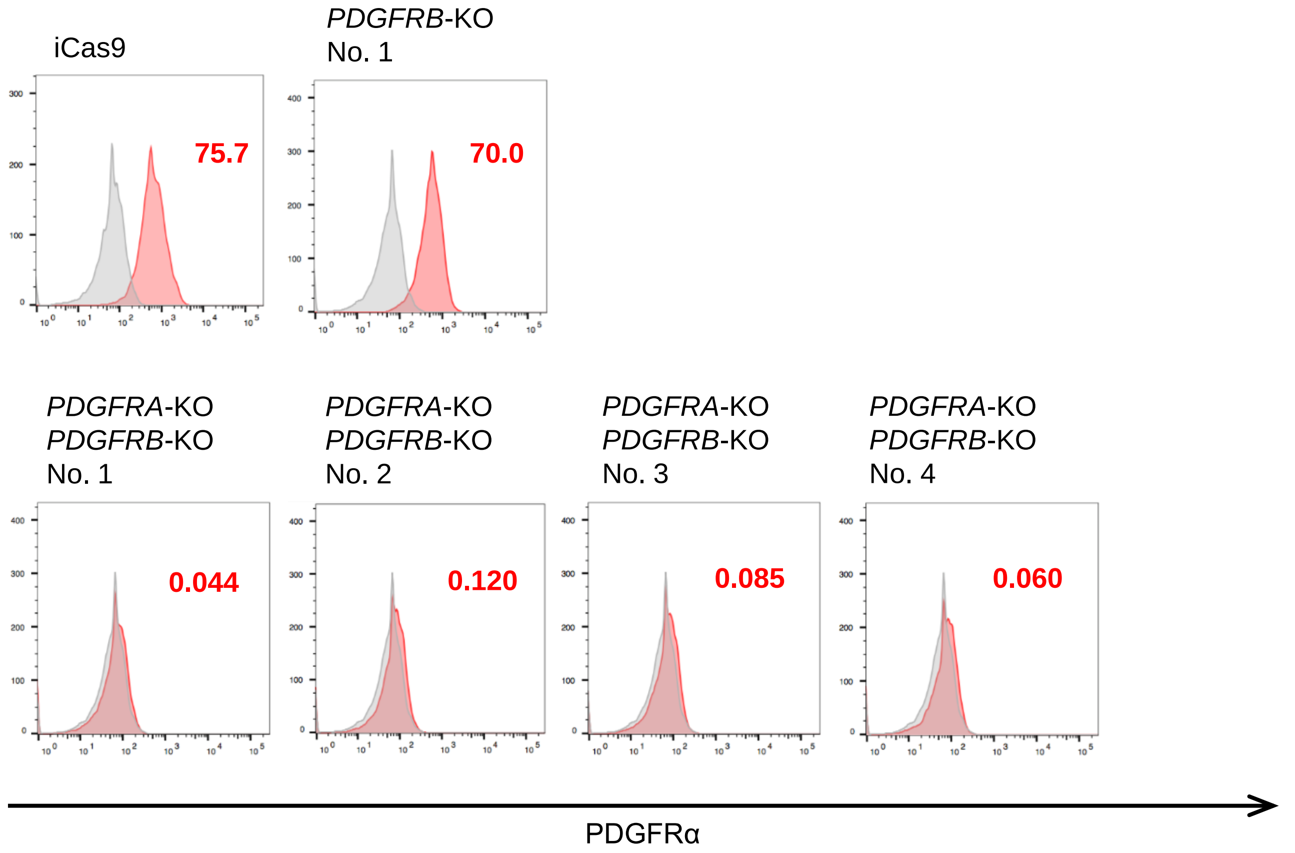

Figure S6. Validation of *PDGFRA/B* double KO cell lines, related to Figure 4.
(A) Sequence analysis of *PDGFRA* gene locus in *PDGFRA/B* double KO cell lines. *PDGFRA/B* double KO cell lines originate from *PDGFRB*-KO No. 1. The yellow bases represent a target sequence, the underlined bases are protospacer adjacent motif in CRISPR/Cas9 system.
(B) Flow cytometric analysis of PDGFRα in *PDGFRA/B* double KO cell lines. Each cell line was differentiated into HE by the d6DE protocol and used for the analysis. The grey lines are the isotype control, the red lines are the sample stained with PDGFRα Alexa Fluor 647-conjugated antibody, and the numbers represent the positive rate (%).

Figure S7

A

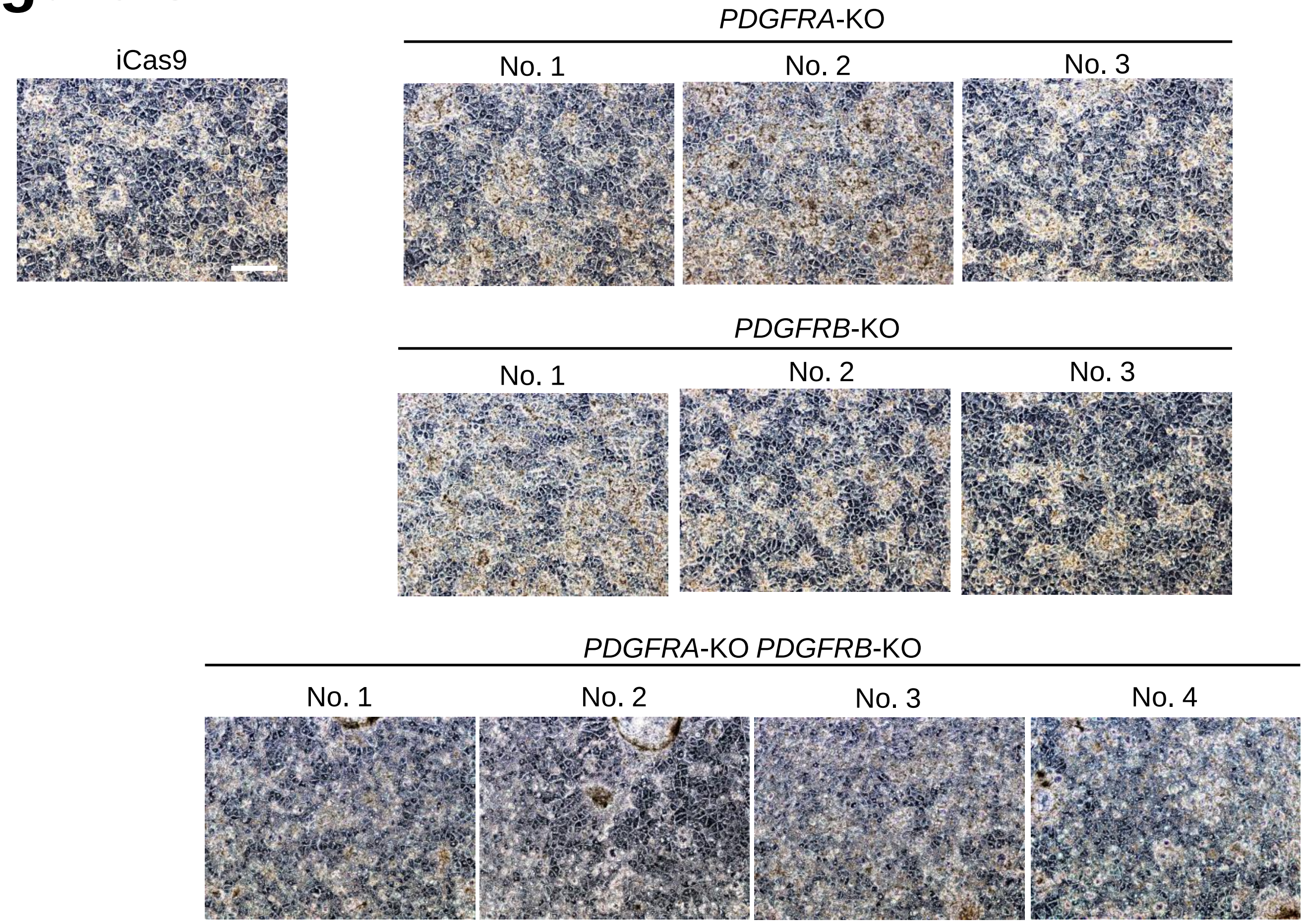

B

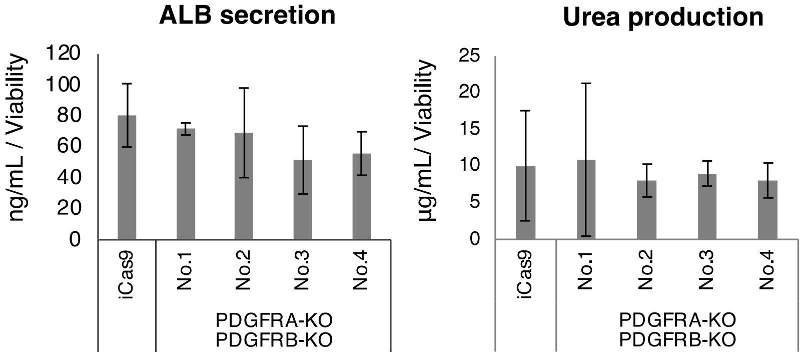

Figure S7. The phenotype analyses of *PDGFRA/B* double KO cell lines in 2D-cultured MH, related to Figure 4.
(A) Morphologies of 2D-cultured MH in iCas9, *PDGFRA*-KO, *PDGFRB*-KO, *PDGFRA/B* double KO cell lines. Scale bar, 200 µm.
(B) Albumin secretion and urea production per cell viability of MH in each cell line. The bars represent mean ± SD. (n = 3).

C

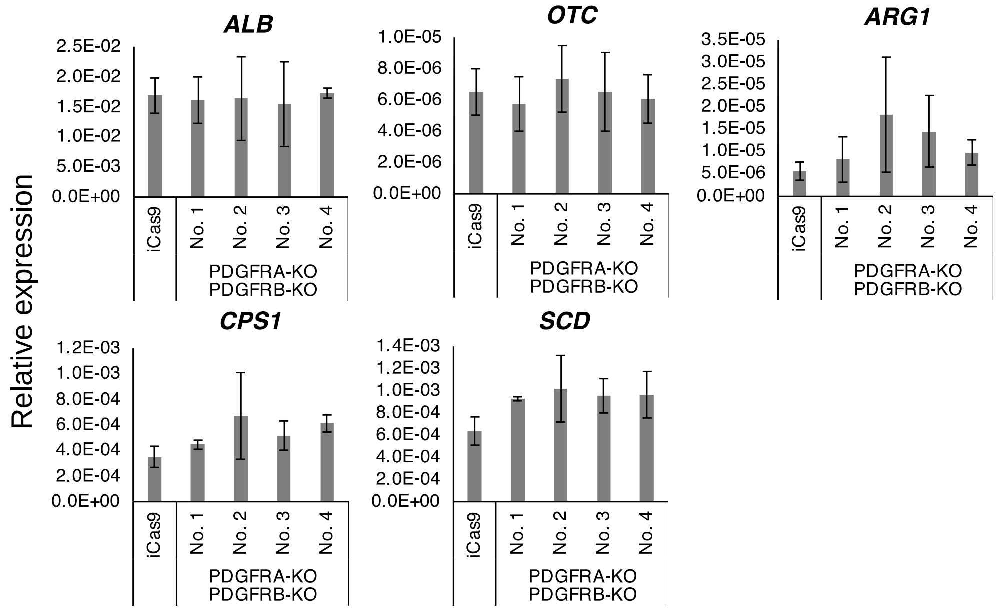

(C) Expression levels of hepatocyte marker genes of MH in each cell line. Normalization is performed using the expression level of 18S rRNA. The bars represent mean ± SD. (n = 3).

Figure S8

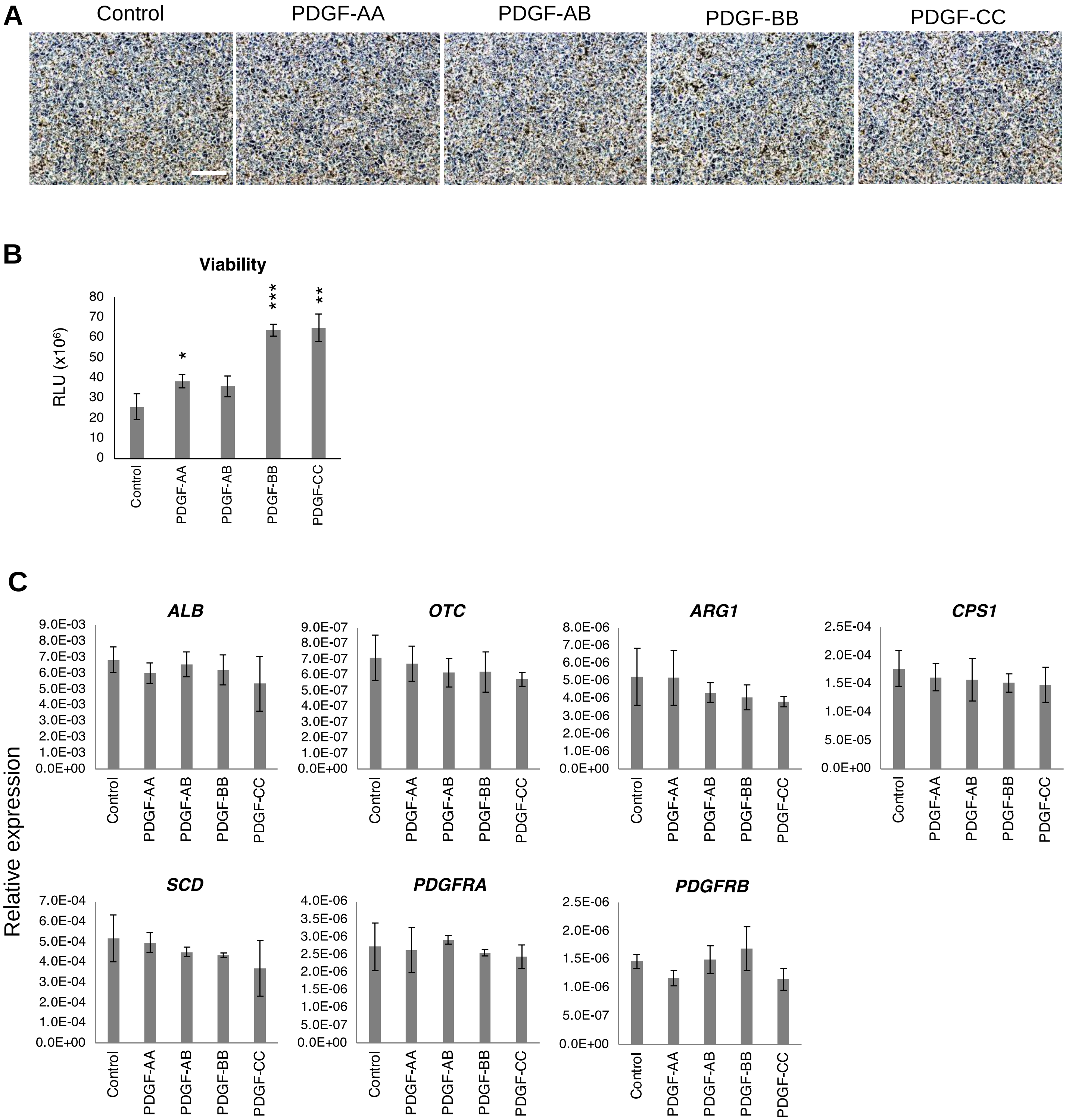

Figure S8. Data of each PDGF-treated 2D-cultured MH, related to Figure 5.
(A) Morphologies of mock control and each PDGF-treated MH. Scale bar, 200 μm.
(B) Cell viability of mock control and each PDGF-treated MH. The bars represent mean ± SD. (n = 3; *p < 0.05; **p < 0.01; ***p < 0.001).
(C) Expression levels of hepatocyte marker and PDGFR genes of mock control and each PDGF-treated MH. Normalization is performed using the expression level of 18S rRNA. The bars represent mean ± SD. (n = 3).

Figure S9

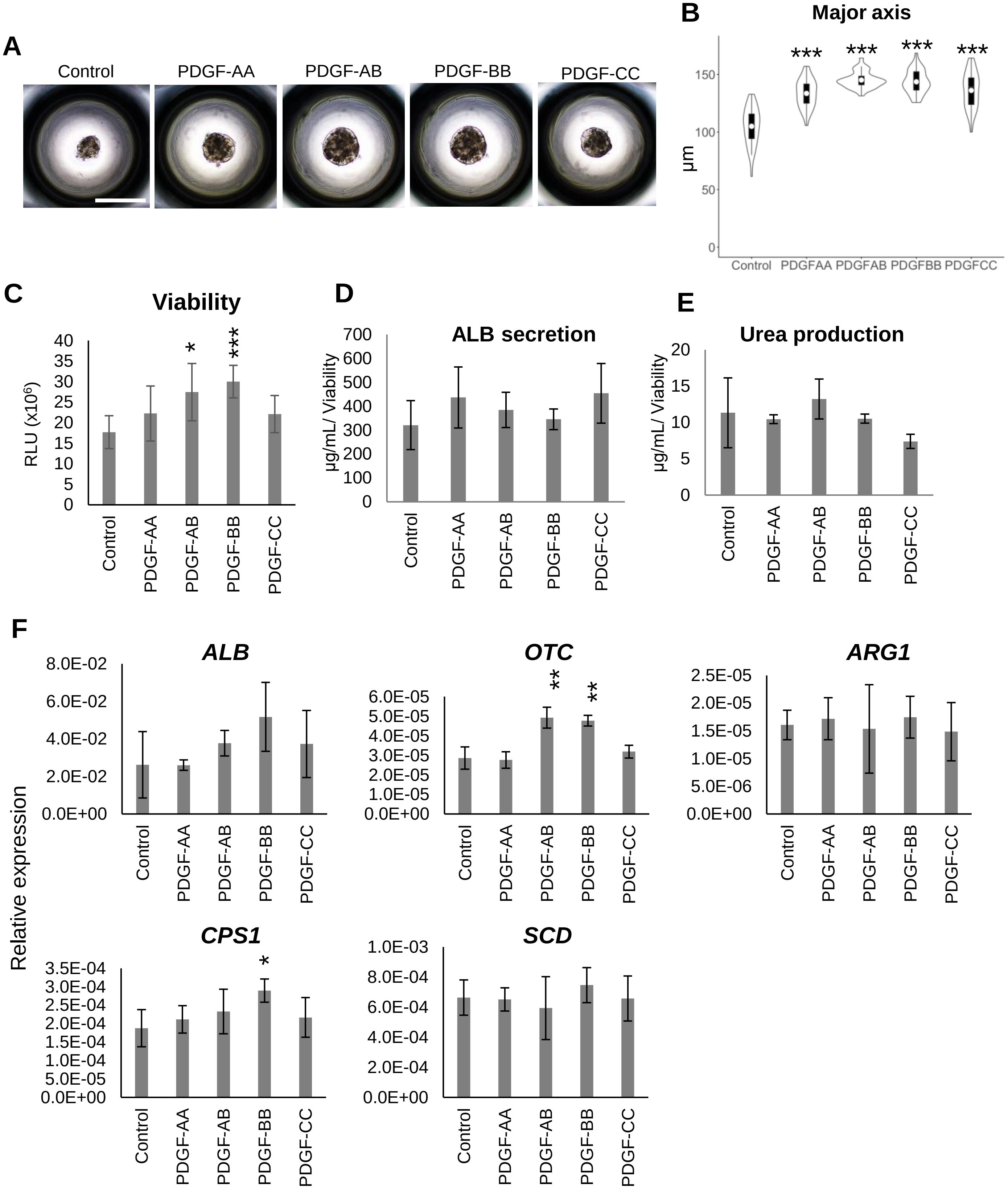

Figure S9. Evaluation of PDGFs effect on HS produced from d6DE day10 cells, related to Figure 5.
(A) Morphologies of mock control and each PDGF-treated HS on day 11 after production. Scale bar, 200 µm.
(B) Violin plot of major axis length of mock control and each PDGF-treated HS on day 11. (n = 30 to 33; ***$p < 0.001$)
(C) Cell viability within HS by measuring intracellular ATP levels in each HS. The bars represent mean ± SD. (n = 6; *$p < 0.05$; ***$p < 0.001$). RLU: relative luminescent unit.
(D) Albumin secretion per cell viability in d each HS. The bars represent mean ± SD. (n = 3; *$p < 0.05$; **$p < 0.01$).
(E) Urea production per cell viability in each HS. The bars represent mean ± SD. (n = 3).
(F) Expression levels of hepatocyte marker genes in each HS. Normalization is performed using the expression level of 18S rRNA. The bars represent mean ± SD. (n = 3; *$p < 0.05$; **$p < 0.01$).

Figure S10

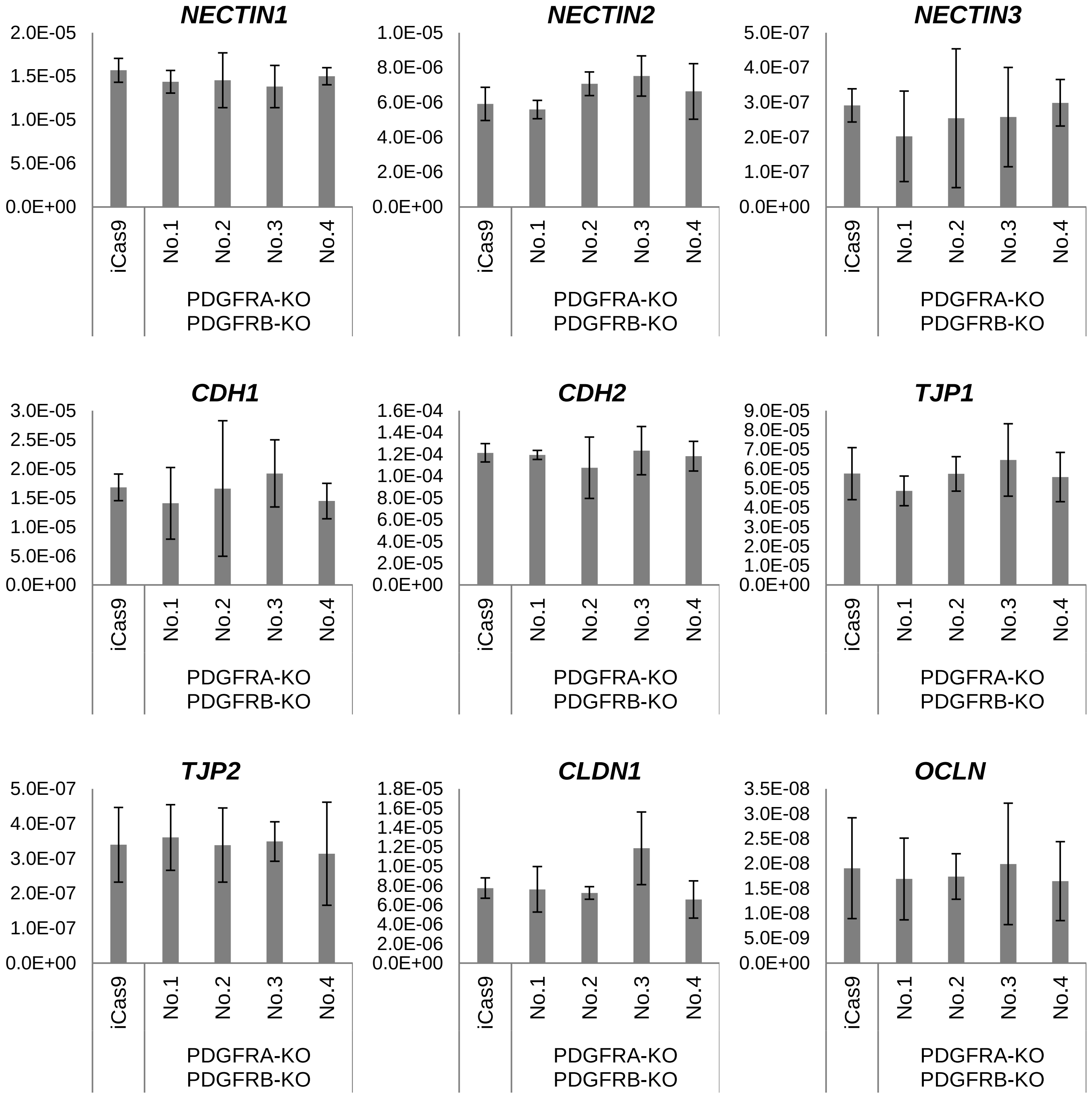

Figure S10. Expression levels of major cell adhesion molecules on d6DE day 10 cells, related to Figure 6. Normalization is performed using the expression level of 18S rRNA. The bars represent mean ± SD. (n = 3).

Table S1: Genes encoding cell membrane protein screened by Comparison 1 to 4

Gene symbol	Comparison 1 FC	Comparison 2 FC	Comparison 3 FC	Comparison 4 FC
PDGFRA	6.95	4.61	3019.15	1757.08
SEMA7A	33.95	5.09	18.83	1.55
EDARADD	7.48	36.41	3.32	4.25
TMEM37	10.67	4.22	27.16	11.25
SLC39A4	9.54	4.91	10.46	14.86
SLC7A11	4.14	12.89	2.91	2.39

FC: fold change.

Table S2: Antibodies used in this study.

Antibody	Vender	Catalog No.
Anti-human FOXA2	Millipore	07-633
Anti-human SOX17	R&D SYSTEMS	AF1924
Anti-human NECTIN3	Millipore	MABT63
Anti-human NECL5	Millipore	MABF2245
Anti-human E-cadherin	R&D SYSTEMS	MAB1838
Anti-mouse HNF4A	Thermo Fisher Scientific	MA1-199
Anti-mouse PDGFRα	R&D SYSTEMS	AF1062
Anti-mouse NECL5	R&D SYSTEMS	MAB6909
Anti-mouse NECTIN3	abcam	ab63931

Table S3: Primers and probes used for qRT-PCR in this study.

Gene	Forward	Reverse	Probe No.
CER1	GCCATGAAGTACATTGGGAGA	CACAGCCTTCGTGGGTTATAG	41
CXCR4	ATTGGGATCAGCATCGACTC	CAAACTCACACCCTTGCTTG	79
TBX3	GCAGCTTTCAACTGCTTCG	ACCCTCGCTGGGACATAAAT	19
HNF4A	TCAGACCCTGAGCCACCT	AGCAACGGACAGATGTGTGA	27
AAT	AATGGGGCTGACCTCTCC	GTCAGCACAGCCTTATGCAC	82
ALB	AATGTTGCCAAGCTGCTGA	CTTCCCTTCATCCCGAAGTT	27
APOA1	CCTTGGGAAAACAGCTAAACC	CCAGAACTCCTGGGTCACA	39
ASGR1	TGACCACCATCAGCTCAGAA	ACAGACAACCACAAGCAGCA	8
ARG1	CAAGGTGGCAGAAGTCAAGA	GCTTCCAATTGCCAAACTGT	64
CPS1	ATTCCTGCACAAGTGGACCT	TGACTCGGAAGCTGCTCAC	8
FGA	GGAAATTTTGAGAGGCGATTT	CCTCTGACACTCGGTTGTAGG	30
SCD	CCTAGAAGCTGAGAAACTGGTGA	ACATCATCAGCAAGCCAGGT	82
OTC	CAGCGAAATTCGGAATGC	CTAGCATCCGGCTCATAACC	27
PDGFRA	CCACCTGAGTGAGATTGTGG	TCTTCAGGAAGTCCAGGTGAA	27
PDGFRB	CATCTGCAAAACCACCATTG	GAGACGTTGATGGATGACACC	10
SEMA7A	CCTTTCATGTGCTTTACCTAACTACA	GATGTTGAAGGCGAAGCTGT	15
EDARADD	ACTGCCCACGAAATTCAGATA	TGGAAGAGGATCTCCAGTGC	7
TMEM37	CCCAGGGTTGTTAAGAATGG	GAAAGTCCTAGGCACTGATTGG	11
PDGFA	GATGAGGACCTTGGCTTGC	CCAGCCTCTCGATCACCTC	68
PDGFB	CTGGCATGCAAGTGTGAGAC	CGAATGGTCACCCGAGTTT	68
PDGFC	CAGCAACAAGGAACAGAACG	TGGGCTGTGAATACTTCCATT	73
PDGFD	ACCATGACCGGAAGTCAAAA	CTGGGAGTGCAACTGTAACG	85
CDH1	AGGGGTCTGTCATGGAAGGT	GCGGCATTGTAGGTGTTCA	5
CDH2	GGTGGAGGAGAAGAAGACCAG	GGCATCAGGCTCCACAGT	66
NECTIN1	GACTCGCTCTCGGCTTGA	CGTTCACCTGGACCACCT	69
NECTIN2	GAGGACGAGGGCAACTACAC	TTGGGCTTGGCTATGACTCT	60
NECTIN3	TGACCAAAAAGTCATCTACATTTCA	TTGAGGGATGCCACTGAAT	41
TJP1	CCAGCTGGTATGGGTTTCC	TCTACTGTCCGTGCTATACATTGAGT	1
TJP2	GAGGCGCCTACACTGACAAT	TTCTGGGCAATTTCGATCTC	42
CLDN1	TTGACTCCTTGCTGAATCTGAG	GGCCACAAAGATTGCTATCAC	79
OCLN	GTCATCCAGGCCTCTTGAAA	AATGGCAATGGCAATTCATC	10
